# Associations of water quality with cholera in case-control studies: a systematic review and meta-analysis

**DOI:** 10.1186/s12879-025-11533-x

**Published:** 2025-09-26

**Authors:** Thuy Tien Nguyen, Chaelin Kim, Gerard Goucher, Jong-Hoon Kim

**Affiliations:** 1 Institute for Medical Information Processing, Biometry, and Epidemiology - IBE, LMU Munich, Munich, Germany; 2 Pettenkofer School of Public Health, Munich, Germany; 3 International Vaccine Institute, Seoul, South Korea

**Keywords:** Cholera, Water, sanitation, and hygiene, Case-control, Meta-analysis, Odds ratio

## Abstract

Cholera is a significant health risk for low- and middle-income countries (LMIC), and the threat of outbreaks is likely to increase due to climate change. To keep up to date with the link between water quality and cholera, we conducted a systematic review and meta-analysis to update a previous review while only focusing on water-related exposures. We searched Embase, Web of Science and PubMed for literature published between 2016 and 2022. Search terms were consistent with the previous review. Study quality was assessed using the Risk Of Bias In Non-randomized Studies - of Exposures (ROBINS-E). Water-related exposures were categorized according to the WHO/UNICEF Joint Monitoring Program for Water Supply, Sanitation and Hygiene (JMP) and further divided by the service ladder. Odds ratios were extracted and pooled by performing random-effects meta-analysis. We identified 22 new eligible studies and analysed them in addition to the 45 studies included in the previous review. Analyses revealed higher odds of cholera when consuming sachet water (OR = 1.69, 95% CI: 1.13 to 2.52), unimproved water (OR = 2.91, 95% CI: 1.21 to 7.02), surface water (OR = 3.40, 95% CI: 2.52 to 4.58), and untreated water (OR = 2.51, 95% CI: 2.03 to 3.10). Meanwhile, treating water (OR = 0.42, 95% CI: 0.27 to 0.65) by boiling (OR = 0.38, 95% CI: 0.17 to 0.84) or chlorination (OR = 0.37, 95% CI: 0.17 to 0.83), and drinking basic water (OR = 0.44, 95% CI: 0.27 to 0.69) showed protection. Water-related exposures were prioritized to synthesize the evidence on cholera transmission through drinking water, with sanitation and hygiene as additional risk factors. While the JMP service ladder provided a useful classification framework, context-specific differences in infrastructure and interpretation should be considered when evaluating these findings. Pooled estimates changed with updated evidence while qualitative insights on the protective or risk factors remain valid. Relatively low-cost methods like boiling or chlorinating water provide good protection comparable to providing basic water to the public.

**Supplementary Information:**

The online version contains supplementary material available at 10.1186/s12879-025-11533-x.

## Strengths and limitations of this study

- The study updates a previous review, incorporating recent case-control studies (2016–2022), thereby providing updated insights on the association between water quality and cholera.
- The study used the ROBINS-E tool for quality assessment to ensure a systematic and thorough evaluation of studies.
- The exclusive focus on case-control studies introduces potential recall bias and may limit the comprehensiveness of the findings.

## Introduction

Cholera is an acute diarrhoeal disease caused by the bacterium *Vibrio cholerae*. The disease is mainly transmitted through the faecal-oral route by consuming contaminated water or food, or through person-to-person contact [1]. Cholera continues to pose a major global health challenge, particularly in low- and middle-income countries (LMICs), where inadequate sanitation infrastructure and limited access to clean water exacerbate the risk [2]. In 2022, more than 29 countries reported new outbreaks to the World Health Organization (WHO), with Lebanon and Syria being considered cholera non-endemic countries [2]. Major outbreaks often occur in areas of humanitarian crisis, political instability, and water insecurity [2]. This was illustrated by the 2016 outbreak in Yemen, one of the largest cholera epidemics in recent times as a result of ongoing armed conflict [3].

Accurately estimating the global burden of cholera is difficult. While the World Health Organization (WHO) reported 323,320 cholera cases and 857 deaths in 2020, these figures were likely to be considerably underestimated [4]. Factors such as inadequate surveillance systems, fears of negative economic impacts on trade and tourism, lack of diagnostic tools, and the recent COVID-19 pandemic may have contributed to the underreporting of cholera cases [5]. A modelling study from 2015 estimated the cholera burden to be between 1.3 and 4.0 million cases annually in cholera-endemic countries [6]. The burden may increase with climate change putting potentially even high-income countries at risk in the future [7, 8]. With changing rainfall patterns, recurrent flooding, and rising temperatures, the risk of faecal-oral pathogens contaminating the environment is increasing. This threatens water quality and increases the transmission of waterborne diseases especially in countries with fragile health and sanitation infrastructure [7, 8]. Large and deadly cholera outbreaks have already been observed consequently [2].

The main risk factors for cholera outbreaks are inadequate water quality, poor sanitation and hygiene, and overcrowding [1]. These risks can be counteracted by implementing water, sanitation, and hygiene (WASH) interventions. WASH interventions are one of the most important factors in limiting and preventing outbreaks [9, 10]. The importance of WASH was emphasized by the establishment of the WHO/UNICEF Joint Monitoring Program (JMP) for Water, Sanitation and Hygiene in 1990. The JMP monitors the global progress on WASH towards achieving the Sustainable Development Goals [11]. It regularly collects national and regional-level WASH estimates for more than 190 countries. Data are being reported for three different WASH categories of drinking water, sanitation, and hygiene. Drinking water and sanitation each comprise five subcategories, while hygiene includes three subcategories with information collected across various settings, such as households, schools, and health facilities [12, 13]. Thus, understanding the WASH association with cholera standardized by JMP categories can provide a way to explore the risk associated with cholera in areas where JMP estimates are available.

Although many have already investigated the association between WASH and cholera [14–17], it is important to regularly update knowledge based on new evidence. A better understanding is crucial to guide decision-making and can lead to more cost-effective adaption of intervention programs and thus strengthen the impact of future cholera prevention programs. To our knowledge, there is currently only one systematic review and meta-analysis that explored the association between WASH exposures and cholera in case-control studies. Wolfe et al. published their study in 2018, evaluating studies published between 1990 and 2016 [17]. The authors included water, sanitation, and hygiene and classified WASH exposures according to the JMP standards [17]. However, the JMP-specific service ladders developed for further grading and specification of WASH categories were not used in the previous review.

While Wolfe et al. examined all three categories of WASH which include water, sanitation, as well as hygiene [17], no systematic review has yet focused exclusively on drinking water related to cholera. Out of WASH, focusing on the association between water and cholera is compelling as water is a basic human need and cholera spreads mainly via unsafe water [1]. Case-control studies are particularly interesting to focus on when studying this association, as our preliminary search revealed that major evidence was generated in this study design, and its consistency is an advantage for meta-analyses.

The aim of this study is to update the existing systematic review and meta-analysis while focusing on the association between water and cholera. This review integrates recent evidence to provide updated insights into the association between water quality and cholera in LMICs, employing a more rigorous assessment of study quality.

## Methods

### Search strategy and eligibility criteria

The development of the systematic review and meta-analysis adhered to the Preferred Reporting Items for Systematic Reviews and Meta-Analyses (PRISMA) [18]. PubMed, Web of Science, and Embase were chosen as databases to systematically identify relevant peer-reviewed articles. The search terms were kept consistent with the previous search terms utilized in Wolfe et al.: (“case control” OR “case-control” AND “cholera”) [17].

Results were restricted to articles published in English between 01 July 2016 and 02 September 2022 since articles published before 01 July 2016 were already included in the Wolfe et al. review [17]. All search records were imported to the software Sciwheel, deduplicated, and further screened according to the following predefined inclusion and exclusion criteria: Case-control studies conducted in low- and middle-income countries (LMICs) were included, while those in high-income countries were excluded to focus on low-resource settings. Cases were individuals diagnosed with cholera, and controls were those without cholera infection. No restrictions were applied to age, gender, or socioeconomic status. Only studies evaluating associations between water, sanitation, and hygiene (WASH) exposures and cholera using odds ratios (OR) were included. Detailed Population, Intervention, Comparison, Outcomes, and Study Design (PICOS) criteria [19] are provided in the study protocol [20].

### WASH Exposure categories with focus on water

The JMP WASH categories were used in order to classify the exposures more precisely by additionally using the JMP service ladder to further differentiate these categories into finer levels [21]. However, as this study focuses on water-related exposures, only the category of water was included for further analysis. In addition to the JMP category drinking water, water treatment, and water management were included to describe water and its handling in more detail and to be consistent with Wolfe et al. [17] (Table 1). Exposures deemed eligible were compared to the JMP service ladder for drinking water. They were subsequently allocated to one of the five subcategories (safely managed, basic, limited, unimproved, surface water) according to its definition given by the JMP [22]. Likewise, exposures were classified as improved water sources based on the definition provided by the JMP [22]. However, as the JMP did not provide specific examples for safely managed, basic, and limited drinking water, we matched the exposures to the definitions provided as closely as possible [22].

**Table 1:** Water related exposures from studies included in the analysis and corresponding JMP service ladder

Water Category	JMP Service Ladder		Definition	Examples from Included Studies
Water Source^*^	Improved	Safely Managed	Improved drinking water that is free from faecal and priority chemical contamination while being accessible on site [23]	Drinking bottled water, municipal tap water, sachet water, piped water
		Basic	Improved water that is collected in less than 30 min [23]	Drinking borehole water, rainwater, tube well water
		Limited	Improved water that is collected in more than 30 min [23]	Drinking tanker water, bladder water, common-source municipal tap water
	Unimproved		Unimproved water from unprotected water sources [23]	Drinking unprotected spring water, unprotected well water
	Surface Water		Unimproved water directly from open-sourced water bodies above the ground [23]	Drinking lake water, river water, stream water
Water Treatment^†^	Treated Water		Any household treatment used to make water drinkable. If further specified, then allocated to treated by boiling, chlorination, presence of treatment materials or treatment itself was confirmed by observation of a surveyor. If not further specified, then allocated to generally treated water	Treating water by e.g., boiling or chlorinating
	Untreated Water		No household treatment used to make water drinkable	Drinking untreated water
Water Management^†^	Safe Water Storage		Any method of storing potable water in containers with a lid, a narrow mouth, or a similar design	Storing water in container with lid and/or container with narrow opening
	Unsafe Water Storage		Any method of storing potable water with open containers without a seal or lid or with a wide opening	Storing water in container with wide opening and no covering; changes in colour, odour, and taste of water

^*^ originally named “drinking water” under JMP classification [22]

^†^ categories added to the JMP categories

Water treatment and water management were further divided into treated water and untreated water as well as safe water storage and unsafe water storage, respectively.

Additionally, a more differentiated classification was introduced for similar exposures of the same category. An example would be the differentiation of boiled water and chlorinated water, rather than analysing them jointly as treated water or having to exclude one exposure. Additional inclusion criteria were developed if this differentiation was insufficient to avoid unit-of-analysis error and ensure that certain populations were not overrepresented in the meta-analyses.

### Data extraction

In addition to the articles selected based on the previous review [17], the title and abstract of the newly identified articles were screened and checked for relevance by two independent researchers (CK and TN), and eligibility was determined in accordance with the PICOS framework. After this initial screening, the full texts of the included studies were further independently examined for eligibility by the same researchers. Discrepancies in both steps were discussed afterwards with a third researcher (J-HK) to reach consensus.

Relevant data were extracted and compiled using Google Sheets. For each article, following information was extracted: the country where the study was conducted, the WASH exposures analysed, the OR and corresponding confidence interval (CI), the general adjustment factors used, and the adjustment for other WASH exposures.

### Risk of bias assessment

To appraise the quality of the eligible studies, we used the Risk Of Bias In Non-randomized Studies - of Exposures (ROBINS-E) tool, which is specifically designed to assess the risk of bias in estimates from observational studies of exposures on health outcomes [23]. The tool assesses the risk of bias in seven different domains: (1) confounding, (2) measurement of exposure, (3) selection of participants, (4) post-exposure interventions, (5) missing data, (6) measurement of outcome, and (7) selective reporting of results. Each domain was graded on a scale of “low risk of bias”, “some concerns”, “high risk of bias”, and “very high risk of bias” which was subsequently summarized in an overall risk of bias. Studies scoring “low risk” in more than one domain were categorized as overall “some concerns”; studies scoring “some concerns” in more than five domains were considered “high risk”; and those scoring “high risk” in more than one domain were categorized as “very high risk”. Consistent with ROBINS-E guidance [23], we tailored the overall risk-of-bias algorithm based on domain rating patterns in our dataset. The selected threshold balances the exclusion of biased studies with the need to retain sufficient evidence for meta-analysis. The assessment of quality was performed by two reviewers independently (CK and TN) and then discussed in conjunction with the third researcher (J-HK).

### Statistical analysis

Studies labelled as “very high risk” during the quality assessment were excluded from the analysis. Exposures that were identified by the included articles as contaminated and potentially even the origin of an outbreak were excluded. This was due to the high likelihood of bias in the meta-analysis as a result of misclassification of an exposure that was originally confirmed to be contaminated. In addition, only one exposure per reporting unit was included in each analysis to avoid unit-of-analysis error and to ensure that no study population was given more weight in the analysis. This was achieved by choosing exposures with narrower confidence intervals, and thus more precise estimates, over similar exposures with wider intervals. In case of similar width of confidence intervals, preference was given to exposures that were more comparable to other exposures in the same subcategory or subgroup. For example, if an article examined the exposure of “drinking river water” and “drinking swamp water” in the same reporting unit, “drinking river water” was included in the analysis and “drinking swamp water” was excluded. The reason was that “drinking river water” was more comparable to the exposures included in other articles in that subgroup. Also, confidence intervals had to be reported in the study to be included in the statistical analysis. Exposures with p-values only were excluded.

The statistical programming language R (version 4.2.2) [24] with the *metafor* package (version 3.8.1) [25] was utilized to conduct the meta-analysis. Meta-analysis was performed for categories with at least two studies with the potential of meaningful pooling [26]. Adjusted ORs were preferred over crude ORs; if not given, matched ORs were utilized. If neither the adjusted OR nor the matched OR were reported, the crude OR was used for meta-analysis. Random effects models were chosen to fit the data over the fixed effects model since real differences in the effect sizes, instead of sampling error, were suspected because of the variability in the setting and the population studied, as indicated by high *I*² (Table 2). The heterogeneity between studies was estimated by restricted maximum likelihood [27] and calculated using τ^2^, Cochran’s Q*-*statistic for subgroup interactions [28], and I^2^-statistic [29]. An overall summary estimate with confidence interval was calculated for each JMP subcategory and two additional categories: “safely managed water” and “treated water”. Publication bias was primarily inspected by examining the symmetry of the funnel plots and calculating Egger’s regression [30].

**Table 2:** Results of the Meta-Analysis

Indicator of water quality	*N *exposures	Pooled OR (95% CI)	Test of Heterogeneity			Wolfe et al. (2018) OR (95% CI)
			Cochran’s Q	τ^2^	I^2^ (95% CI)	
Improved water						1.08 (0.54–2.15)
Safely managed water						
Sachet water	3	1.69 (1.13–2.52)	3.505	0.000	0.00 (0– >99.56)	
Bottled water	5	0.52 (0.19–1.36)	27.126**	1.017	83.01 (53.62–97.80)	0.35 (0.13–0.96)
Tap water	11	0.92 (0.41–2.08)	101.537**	1.670	90.42 (79.65–96.84)	
Basic water	5	0.44 (0.27–0.69)	7.460	0.008	2.49 (0.00–98.12)	
Limited water	4	0.84 (0.14–4.89)	15.874**	2.773	88.81 (58.13–99.33)	
Unimproved water	9	2.91 (1.21–7.02)	52.609**	1.437	88.67 (71.92–97.49)	3.42 (2.47–4.74)
Surface Water	10	3.40 (2.52–4.58)	9.026	0.000	0.00 (0.00–75.97)	2.27 (1.07–4.80)^a^
Treated water	9	0.42 (0.27–0.65)	19.214*	0.231	54.21 (5.73–87.98)	0.44 (0.35–0.56)
Boiled	7	0.38 (0.17–0.84)	24.006**	0.899	81.52 (52.16–96.98)	
Chlorinated	6	0.37 (0.17–0.83)	19.943**	0.736	78.95 (40.66–96.66)	
Observation of Treatment Materials	4	0.41 (0.15–1.14)	12.170**	0.763	75.00 (19.80–98.15)	
Untreated water	15	2.51 (2.03–3.10)	8.442	0.000	0.00 (0.00–34.40)	3.47 (2.76–4.35)
Safe water storage	6	0.19 (0.03–1.36)	38.199**	4.754	87.19 (63.92–97.62)	0.55 (0.39–0.80)
Unsafe water storage	5	1.32 (0.57–3.03)	20.194**	0.668	77.76 (37.99–97.08)	2.79 (2.13–3.65)

*p < 0.05

**p < 0.01

^a^ categorized as surface water contact by Wolfe et al. [17]

## Results

### Study selection

**Fig. 1:**
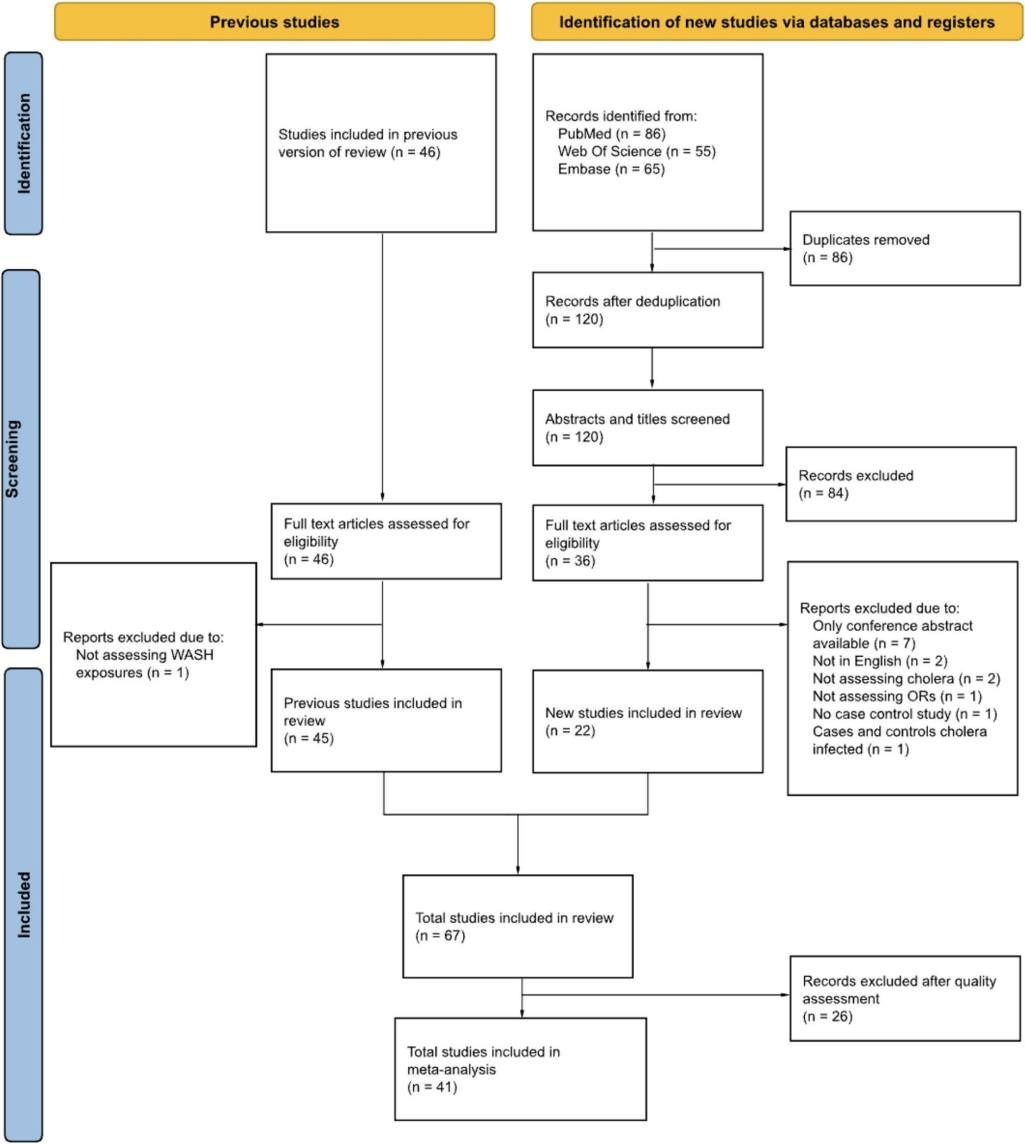
PRISMA Flow Diagram

The search yielded a total number of 206 articles consisting of 86 articles from PubMed, 55 from Web of Science, and 65 from Embase published between 01 July 2016 and 02 September 2022 (Fig. 1). After deduplication, 120 unique articles were left for title and abstract review. This subsequently resulted in 36 articles that were deemed eligible for full-text screening. During the full-text screening, 14 articles were further excluded (Fig. 1). Wolfe et al.. identified 46 studies in their review, which we also included [17]. During the evaluation of these articles, we noticed that one study focused on food exposures rather than WASH exposures. This article was therefore excluded. Ultimately, 22 new studies, in addition to 45 articles from the previous review of Wolfe et al., were included in this review. A total of 67 studies were then further assessed for quality.

### Characteristics of included studies

The newly identified studies were primarily conducted in Kenya, Uganda, and Ethiopia as well as in Yemen, Nigeria, Zambia, Vietnam, India, Ghana, and the Democratic Republic of the Congo [31–52]. The majority was conducted in Uganda with a total of six studies [37, 38, 43, 47, 48, 50], whereas the studies from the previous review were from 21 different countries with the majority of them conducted in Ethiopia, Haiti, India, Kenya, Malawi and Peru [53–97]. The combination of new and previously identified studies resulted in a wider geographical representation compared to the previous review.

The diagnostic methods used to fully identify cholera cases were by culturing stool samples (39.7%), culturing rectal swabs (14.7%), collecting rectal swabs as well as stool cultures (14.7%), rapid test (7.4%), and rapid test as well as stool culture (2.9%). Only one study additionally collected blood samples and tested for antibody titres for cases as well as controls [97]. Two studies only collected blood samples for controls [84, 93] and one study only for a subset of controls [54]. A total of 20.6% of the included studies did not perform culture confirmation of their cases but rather used pre-defined case definitions. However, these case definitions varied from study to study. Detailed descriptions of the characteristics of each study included can be found in Table S1.

### Risk of bias assessment

Overall, the quality assessment with ROBINS-E resulted in 14 studies (20.9%) being classified as “some concerns”, 27 studies (40.3%) as “high risk”, and 26 studies (38.8%) as “very high risk” (Supplemental Figure S1). The studies classified as “very high risk” were omitted from meta-analysis. Therefore, a total of 41 studies were included in the meta-analysis (Fig. 1). Of the 45 previously included studies, 20 were excluded after our risk of Bias assessment, while the remaining 25 were included in the meta-analysis. A detailed description of the summarised results of the risk assessment can be found in Supplemental Figure S1. No publication bias was detected by inspecting the funnel plots (Supplemental Figures S4-S8) and Egger’s test (Supplemental Table S2).

### Results of synthesis

A summary of the results of the meta-analysis can be found in Table 2.

#### Water source

**Fig. 2:**
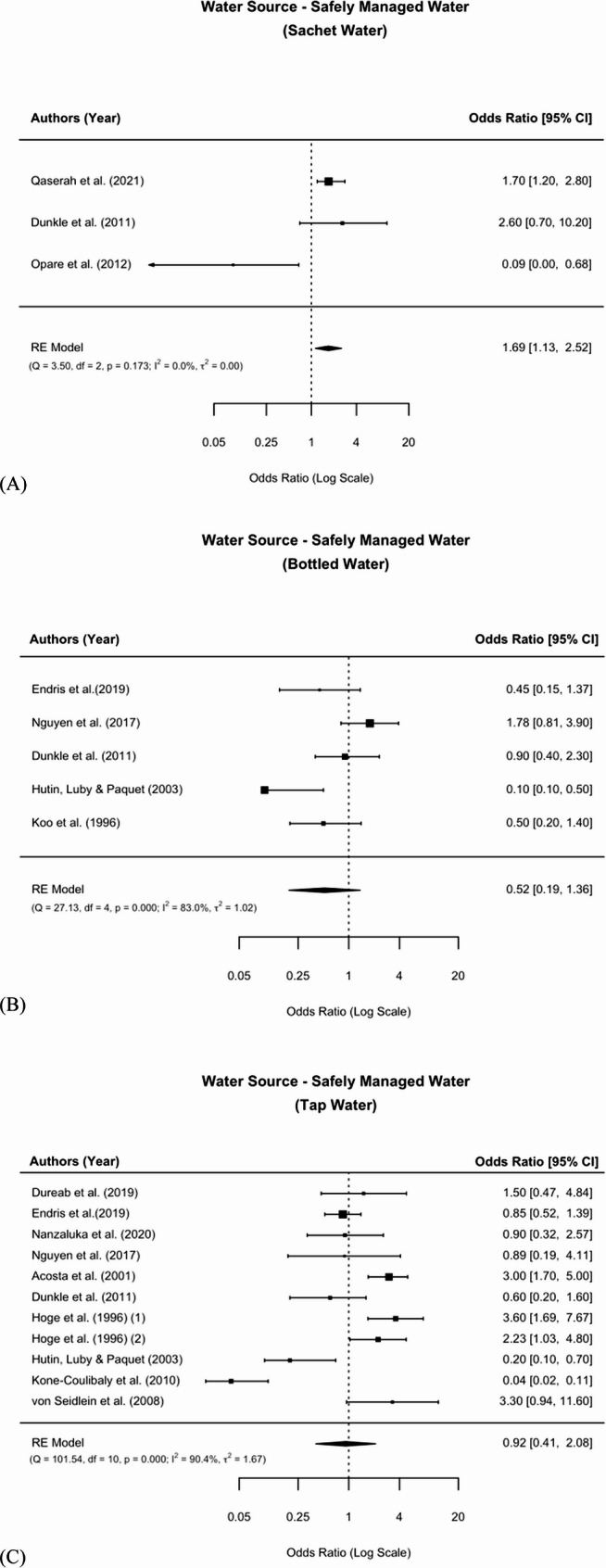
Meta-analysis of the associations between the subgroups of safely managed water and cholera

A total of 21 exposures met the definition of safely managed water and were therefore further subdivided into sachet water, bottled water, and tap water. Sachet water consumption (*n* = 3) was associated with 1.69 times higher odds in cholera (OR = 1.69, 95% CI: 1.13 to 2.52) (Fig. 2). Bottled water (*n* = 5) was protective against cholera, but not statistically significant (OR = 0.52, 95% CI: 0.19 to 1.36). Similarly, tap water (*n* = 11) showed no significant association (OR = 0.92, 95% CI: 0.41 to 2.08) (Fig. 2).

**Fig. 3:**
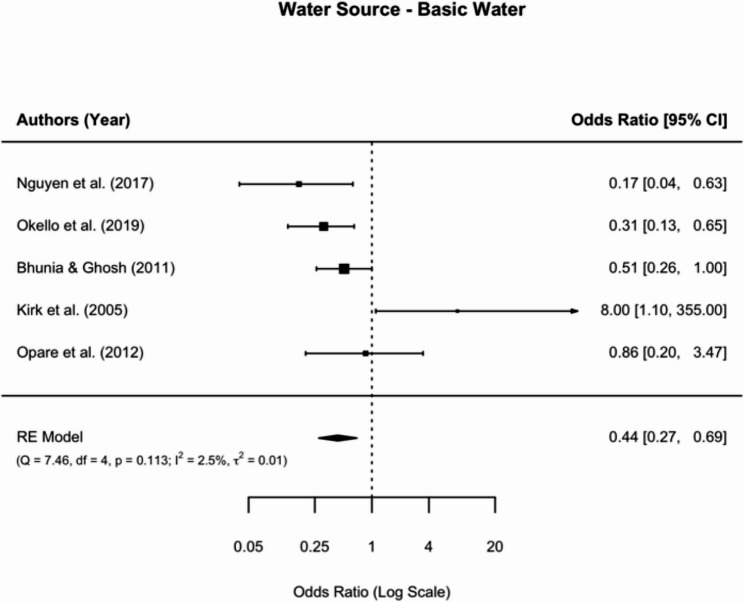
Meta-analysis of the association between basic water and cholera

For basic drinking water sources (*n* = 5), a statistically significant protective association against cholera was observed (OR = 0.44, 95% CI: 0.27 to 0.69) (Fig. 3).

Limited water consumption did not show a significant association with cholera protection (*n* = 4, OR = 0.84, 95% CI: 0.14 to 4.89) (Supplemental Figure S11).

However, the two subcategories, unimproved water as well as surface water, were associated with higher odds of cholera. Consuming unimproved drinking water (*n* = 9) resulted in increased odds of 2.91 (95% CI: 1.21 to 7.02) (Supplemental Figure S12). Similarly, consuming surface water (*n* = 10) was significantly associated with increased odds for cholera, with an OR of 3.40 (95% CI: 2.52 to 4.58), the highest odds for cholera compared to the other subgroups (Supplemental Figure S12).

#### Water treatment

Unspecified water treatment (*n* = 9) was associated with 58% lower odds (OR = 0.42, 95% CI: 0.27 to 0.65) (Supplemental Figure S10). In contrast, untreated water (*n* = 15) significantly increased odds of cholera (OR = 2.51, 95% CI: 2.03 to 3.10) (Supplemental Figure S10).

Similar results were obtained for the subgroups of treated water after synthesis. Boiling (*n* = 7) and chlorination of water (*n* = 6) significantly reduced odds for cholera with OR of 0.38 (95% CI: 0.17 to 0.84) and 0.37 (95% CI: 0.17 to 0.83), respectively (Supplemental Figure S13). No significant protection was detected for having materials for water treatment observed (*n* = 4) (OR = 0.41, 95% CI: 0.15 to 1.14) (Supplemental Figure S13).

#### Water management

Safe water storage (*n* = 6) was associated with a lower OR of 0.19, though not significantly (95% CI: 0.03 to 1.36) (Supplemental Figure S11). Similarly, unsafe water storage (*n* = 5) was with a non-significant OR of 1.32 (95% CI: 0.57 to 3.03) (Supplemental Figure S11).

#### Heterogeneity

Substantial heterogeneity was observed for nearly all subgroups of water source other than consuming sachet water (I^2^ = 0.00%), basic water (I^2^ = 2.49%), and surface water (I^2^ = 0.00%) (Table 2). Similarly, only untreated water was found to have low heterogeneity in the water treatment category (I^2^ = 0.00%). Studies on safe water storage and unsafe water storage revealed that they were substantially heterogeneous, with I^2^ = 87.19% and I^2^ = 77.76%, respectively. Out of 14 conducted meta-analyses, heterogeneity was over I^2^ = 80% in 6 subgroups.

## Discussion

We conducted a systematic review and meta-analysis to examine the association between water quality and cholera. In particular, we updated the previous review by Wolfe et al. [17] by including data from case-control studies published from 2016 to 2022. In comparison with the previous review, the meta-analysis had a more rigorous risk of bias assessment and more stringent inclusion criteria. Our analyses revealed that most of the expected risk factors, such as the consumption of unimproved water and surface water and the lack of water treatment, were associated with higher odds of cholera. Some of the pooled estimates were substantially different from those in Wolfe et al. while qualitative insights were still the same [17]. The exception was unsafe water storage, which failed to show statistical significance after being pooled. A similar lack of statistical significance, but in this case for a protective association, was observed for safe storage of water.

Water treatment and its subgroups showed significant protection against cholera, again in accordance with previous findings of Wolfe et al. [17]. However, the mere presence of equipment used for water treatment was not found to be statistically significant for protection. Contrary to expectations, the only subcategory of improved water that displayed significant protective associations against cholera was basic water. All other subcategories of improved water were not statistically significant. Although Wolfe et al. found a significant protective relationship between drinking bottled water and cholera, our results could not replicate this observation [17]. Moreover, the most striking was that the consumption of sachet water was unexpectedly associated with higher odds of cholera. Furthermore, large discrepancies in between-study heterogeneity and a wide range of observed ORs were reported, depending on the subcategory and subgroup.

Our findings align with Wolfe et al. [17] on the inconsistencies among subcategories of improved water but suggest a more nuanced interpretation. Certain presumed safe water sources, such as municipal tap water or sachet water, may pose higher contamination risks compared to basic or limited water sources lower on the JMP ladder, which, in some contexts, may contribute to cholera outbreaks. We observed several studies in which municipal tap water was associated with higher odds of cholera [34, 53, 66, 96] as well as studies in which tap water was most likely contaminated [41, 57, 85, 91]. The reasons for this were often failures in the piping and sewage systems, which resulted in the tap water being contaminated by, for example, sewage spillages from nearby sewage pipes [41, 52, 57]. In addition, water sold by vendors also appears to be at high risk of contamination. Contamination of packaged water sold on the street does also not appear uncommon, as evidenced by recent findings [41, 44]. Several studies have found significantly high levels of bacterial indicators in sachet water [98–100]. It appears that the level of contamination increases along the supply chain of the sachets [101]. There is evidence suggesting that the water sources used were not the underlying concern, but rather, the packaging and handling of the sachets appeared to be strong indicators of contamination [102]. Given that some case-control studies failed to report protective associations of bottled water [36, 61, 72] or even showed increased odds [46], similar assumptions can presumably be made for bottled water. Packaged water in LMICs often appears to be of questionable quality, as modelling suggests [103]. However, the JMP categorization and its definitions provided by the JMP do not reflect this actual risk and the possibility of contamination (in its service ladder). This discrepancy likely stems from contamination during collection, packaging, or distribution, which the JMP ladder does not account for. This is especially concerning given the growing trend of consuming packaged water in LMICs as a result of water shortages and as an alternative to unimproved water sources [104, 105].

In contrast, the risk factors associated with cholera seem to be consistent and drive outbreaks. The consumption of surface as well as unimproved water tends to confer a constant risk of cholera. A similar pattern can be found in several other studies not exclusively focused on case-control studies [10, 106]. The homogeneity of exposures categorized as surface water consumption may support this. Consumption of surface water appears to be a risk factor regardless of the setting and context of a study. Evidence of surface water being unsafe for drinking and increasing the odds for cholera and outbreaks can be found in past literature [107–109].

Furthermore, our results indicated that treating water with any method implies protection for cholera. Water treatment has the potential to prevent and slow down outbreaks across different LMICs while eliminating the increased risk of cholera from consuming untreated water. This is in good agreement with Cohen and Colford who found a significant protection of boiling water against cholera and several other infectious diseases in similar settings [110]. The mere presence of equipment to treat water did not seem sufficient to provide significant protection. As Lantagne and Yates described in their review, appropriate training for correct and regular usage may be needed to fully take advantage of its protective effect [111].

The lack of a significant effect and the wide observed confidence interval for the either safe or unsafe storage of water could possibly stem from confounding factors such as where the water was collected from as suggested by Birmingham et al. in their case-control study [56]. Other important factors can be whether the water was treated, or the container was cleaned [14]. We observed that none of the exposures included for water management sufficiently controlled for these factors. Other reviews have shown the significance of increased odds of unsafe water management and lowered odds of safe water management [10, 106]. However, it seems that interventions focusing on the impact of safe water management and their implementation in outbreak situations have not been well-described yet and need further research [14].

There were several limitations to our review and meta-analysis that need be considered when interpreting our results. While our analysis specifically focused on water-related exposures, we acknowledge that sanitation and hygiene, key components of the broader WASH framework, were not included. This was a deliberate decision in line with our predefined protocol, aimed at allowing for a more targeted and coherent analysis. Nonetheless, omitting these dimensions represents an important limitation and should be addressed in future systematic reviews seeking a more comprehensive understanding of cholera risk factors. We included only peer-reviewed articles written in English and excluded grey literature and non-English studies. Despite our efforts to reduce publication bias, there may still be a residual risk of publication bias, particularly because of the exclusion of non-English studies. In addition, we observed substantial heterogeneity across several exposure subcategories and subgroups. This was most likely driven by variations in populations, settings (urban vs. rural and outbreak vs. endemic), diagnostic methods, and exposure definitions. While random effects modelling formally accommodates between-study variance, this variability inevitably limits comparability and affects the precision of pooled estimates. Large differences in adjustment for confounders, measurement of exposure, methods of assessment of cases and controls, and contextual factors such as cultural differences, location, and time among the identified studies were most likely influential. Given the diversity of primary studies, it is improbable that all effect-modifying factors were fully accounted for in our meta-analytic models. Leave-one-out sensitivity analysis was not undertaken because several exposure subgroups contained four or fewer studies; removing even one study would have eliminated the pooled estimate. Robustness was instead addressed by (i) excluding studies at critical ROBINS-E risk, (ii) applying random-effects models, and (iii) providing a detailed heterogeneity discussion. We frequently observed a lack of adjustment for confounders in included studies. Most studies did not sufficiently control for the influencing effects of other WASH factors as well as other sources of drinking water, leaving residual confounding likely. Also, the importance of well-defined baseline risk of each exposure to assess ORs cannot be neglected in a study design since they vary across contexts and settings. However, precise descriptions were often missing [36, 46, 61, 72].

Furthermore, the decision to focus only on case-control studies as major evidence on the association between cholera and water inherently results in a certain risk of recall bias due to its retrospective design.

Additionally, we observed that most studies either culture-confirmed only a subset of patients or identified cases solely by clinical definition rather than laboratory testing. Therefore, a certain risk of misclassification must be considered when interpreting these results, given that many cholera cases are asymptomatic [112–114]. Moreover, only a few of the studies that we included assessed the water quality using laboratory testing. Therefore, it was not possible for this review to completely guarantee that the water sources we categorized as safely managed are indeed free of faecal contamination and priority chemicals. However, because many primary reports did not state whether the source satisfied every JMP safely managed criterion, we sometimes inferred ladder placement from incomplete information, which may have led to nondifferential exposure misclassification. Most of the included studies relied on surveys and used this method to assess the exposures. This underlines the importance of regularly assessing the quality of water sources that are supposedly safely managed to ensure water safety. Another factor to consider when interpreting the results is that this review did not differentiate between endemic and epidemic cholera, which may influence the associations.

In addition, we used ROBINS-E for quality assessment and excluded estimates from studies with a very high risk of bias from the meta-analysis, whereas the previous study did not exclude studies based on the quality assessment results [17, 23]. Despite some limitations of ROBINS-E, a widely accepted tool specifically for observational exposure studies, ROBINS-E may be the appropriate option currently available. The use of this tool allowed the identification of studies that appeared to be at very high risk of bias. This resulted in the number of studies and exposures included in each subgroup and subcategory of the meta-analysis being smaller, thereby limiting statistical power in certain exposure categories. This was further compounded by the exclusion of studies that reported only p-values without the confidence intervals required for inclusion in the meta-analysis.

In order to retain a sufficient number of studies to conduct the analysis, we applied a modified definition of studies that were overall categorized as some concerns: Instead requiring two domains, at least five domains had to be rated as some concern to be regarded as an overall high-risk study. Nevertheless, the number of studies classified as having some concerns and the corresponding exposures were insufficient to allow a reasonable meta-analysis to be performed. Accordingly, this resulted in the need to include studies classified as high-risk, which may consequently introduce bias and restrict the generalizability of our pooled estimates. At the same time, it is also important to acknowledge that in outbreak settings, conducting methodologically flawless studies is rarely feasible and high-quality evidence is therefore scarce. Diagnostic constraints illustrate this dilemma: when laboratory confirmation is unfeasible during an outbreak, investigators must rely on clinical case definitions, increasing outcome misclassification and inflating risk-of-bias ratings while reflecting the practical limits of field research. Such constraints make methodologically flawless studies and, by extension perfectly unbiased meta-analytic evidence, difficult to achieve in outbreak settings.

While these are important considerations to keep in mind when interpreting our results, we believe that our review provides a more profound understanding of the association between water quality and cholera. It lays the groundwork for future interventions as well as future research as cholera remains a major public health threat in LMICs. Simple and relatively inexpensive interventions such as water treatment can considerably reduce the burden of cholera infection. While acknowledging that our insights may need contextualization and differentiation for each cholera-affected community before being applied elsewhere, it is evident that regular water treatment can play a crucial role in preventing future outbreaks and should be considered as an early-on containment measurement. This is particularly beneficial where water quality is compromised, and rapid intervention is needed. A holistic approach is essential for future interventions in LMICs: Safe water supply infrastructure must be expanded. It must be made accessible to people in LMICs who are most vulnerable to cholera in order to avoid recourse to water sources with a high risk of infection. Moreover, it may not be sufficient to eliminate risk factors and rely on protective factors as containment measures for outbreaks. Water that is supposedly safely managed may be contaminated and, rather than being protective, may be a driver of outbreaks. Vended water is gaining importance in the face of water shortages [104] making it even more crucial to establish clear regulations to prevent contamination and ensure that even supposedly safe water sources meet stringent safety standards. Furthermore, when planning future interventions, it’s essential to consider the incorporation of cholera vaccines and their potential for medium to short-term protection in conjunction with water safety interventions [105].

## Conclusion

Our findings highlight the urgent need for ongoing efforts to prevent and control cholera, especially in low-resource settings. Although our review concentrated specifically on water-related exposures, these findings should be viewed within the broader WASH framework. Sanitation and hygiene, while beyond the scope of our analysis, are fundamental to cholera prevention and should be systematically explored in future research to complement our water-focused insights. It is critical and essential to continue efforts to prevent and control cholera by ensuring access to safe drinking water, alongside improvements in sanitation and hygiene. Our study further emphasizes the importance of regularly monitoring supposedly safe water, such as packaged or municipal water sources, to ensure they meet safety standards, thereby reducing the risk of outbreaks. It is crucial to make sure the so-called improved water sources, such as sachet water or bottled water, are safe in LMICs, while also expanding services for safely managed drinking water. Simultaneously with the development of safe water infrastructure, addressing the unique and specific contextual challenges in cholera-affected areas, it is recommendable to incorporate relatively simple and inexpensive early protection measures, such as boiling or chlorination, wherever feasible. These measures can serve as effective short-term approaches to prevent and contain future cholera outbreaks in LMICs. Nonetheless, reliance on these interim measures underscores the need for sustained infrastructure investment and community-level behaviour change to achieve durable cholera control.

## Supplementary Information

Supplementary Material 1.

Supplementary Material 2.

## Data Availability

The dataset and R codes for generating figures and tables are available at the GitHub repository of the corresponding author: https://github.com/kimfinale/Cholera_WASH_meta.
