# Supplementary material for: Associations of water quality with cholera in case-control studies: a systematic review and meta-analysis
Source: BMC Infect Dis. 2025 Sep 26;25:1165. doi: 10.1186/s12879-025-11533-x (PMC12465603; doi:10.1186/s12879-025-11533-x)
Supplement: Supplementary file 1 — Supplementary Material 1. [file 12879_2025_11533_MOESM1_ESM.docx]

Figure S1. Risk of bias assessment results


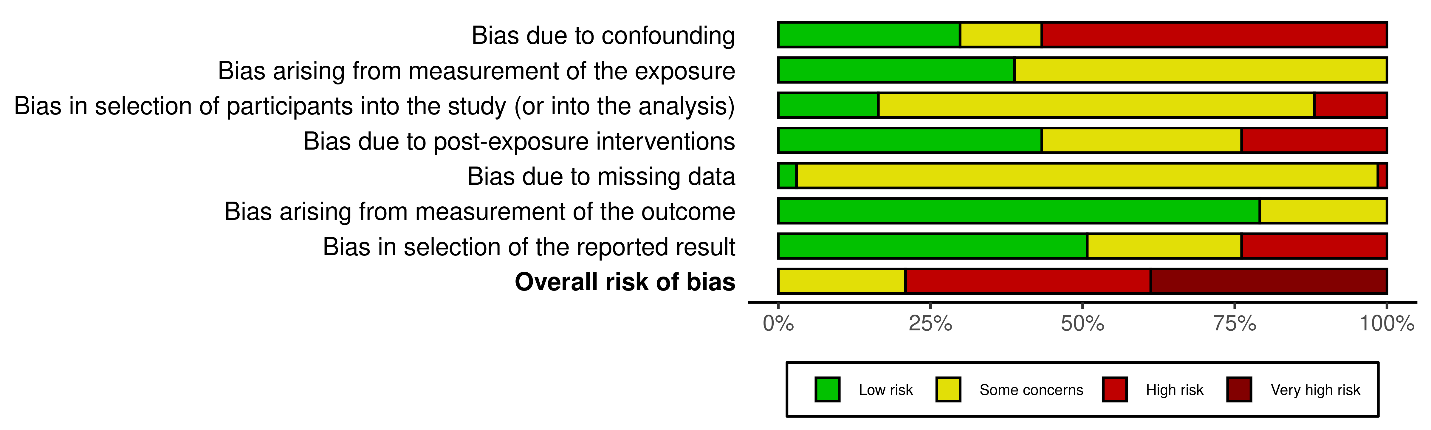


Figure S2. Funnel plots of safely managed water. (A), (B), (C) represent Sachet water, bottled water, and tap water, respectively.

1. (B)


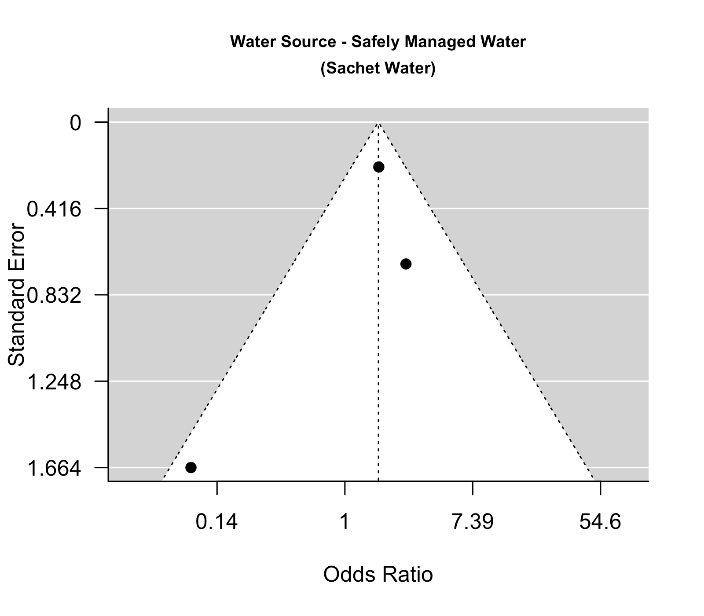

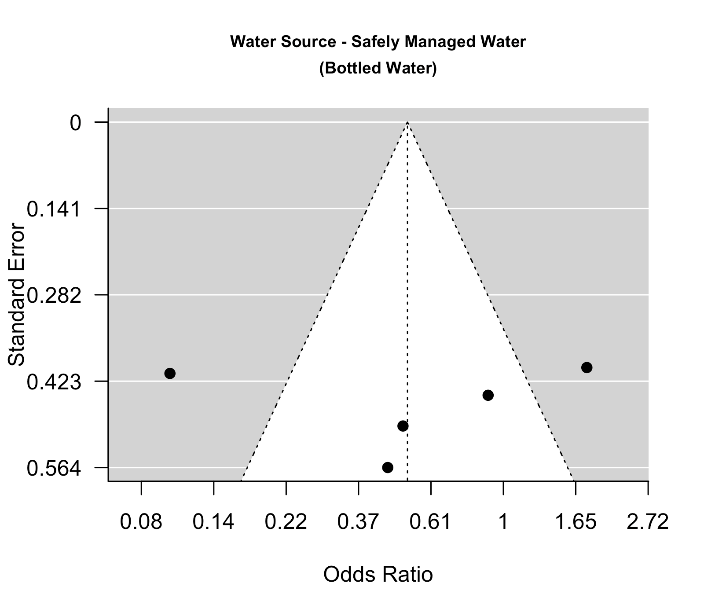


(C)


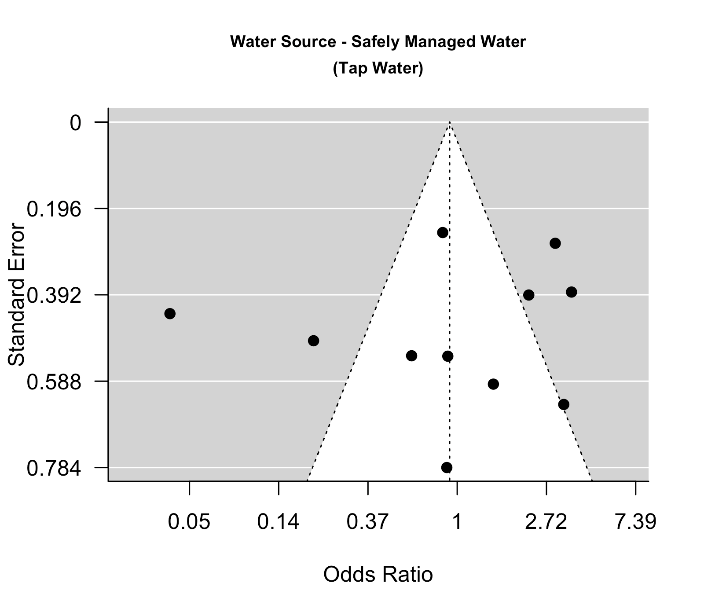


Figure S3. Funnel plots of basic water.


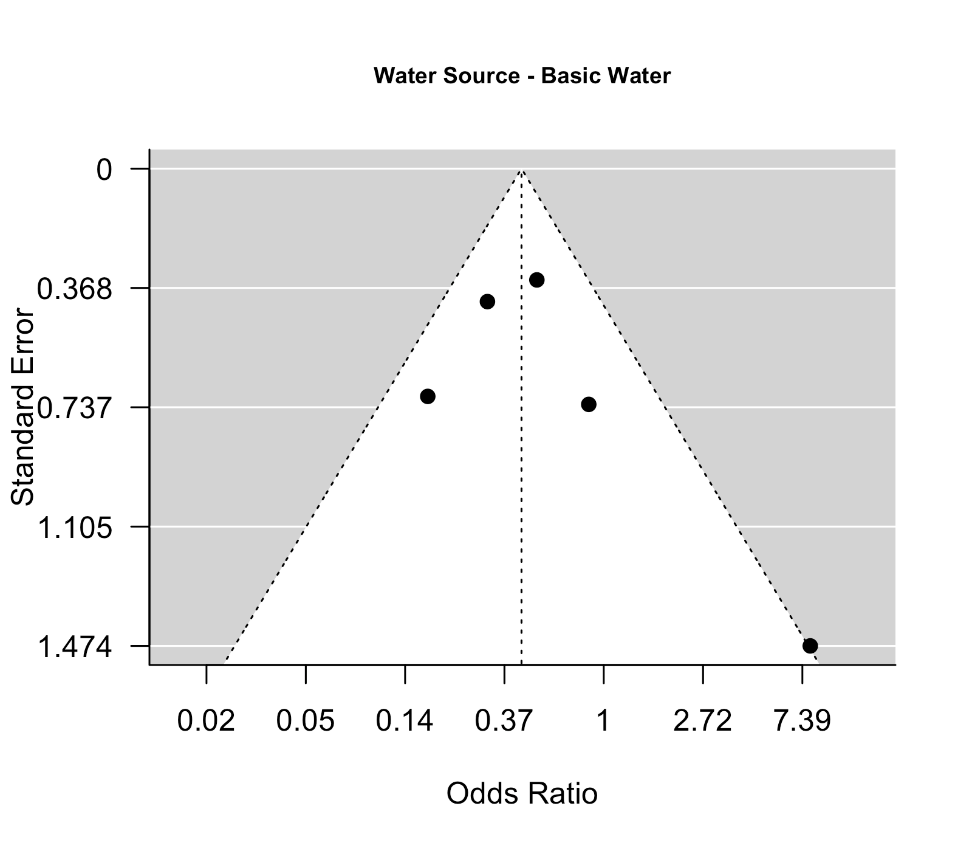


Figure S4. Funnel plots of limited water.


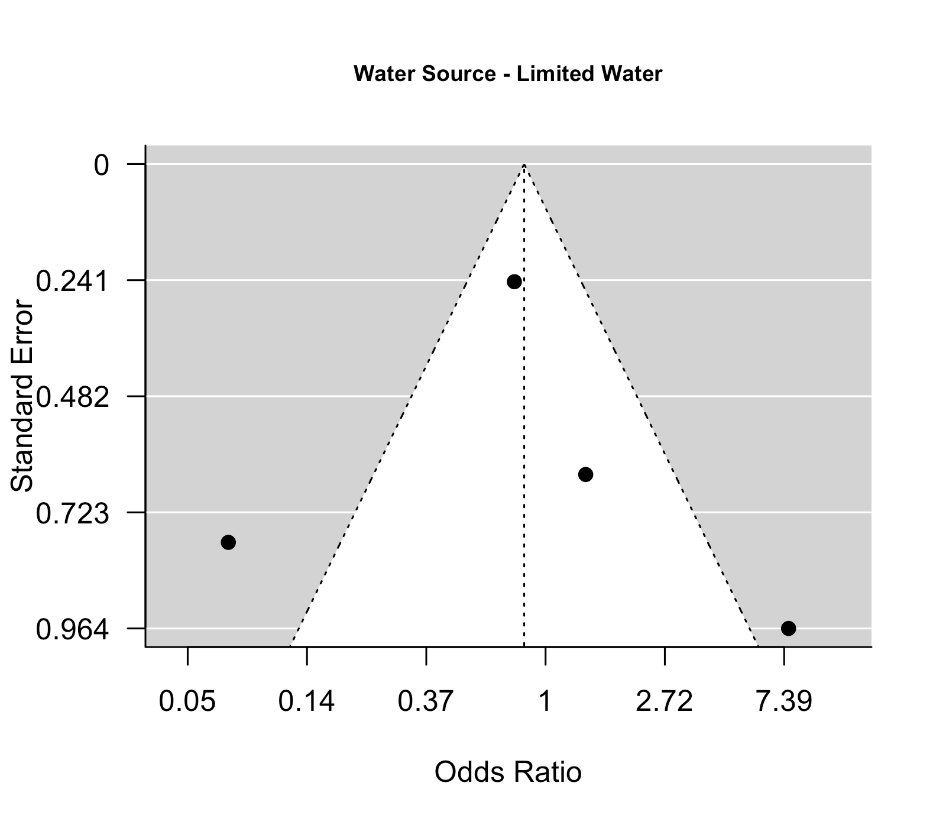


Figure S5. Funnel plots of unimproved water (A) and surface water (B).

1. (B)


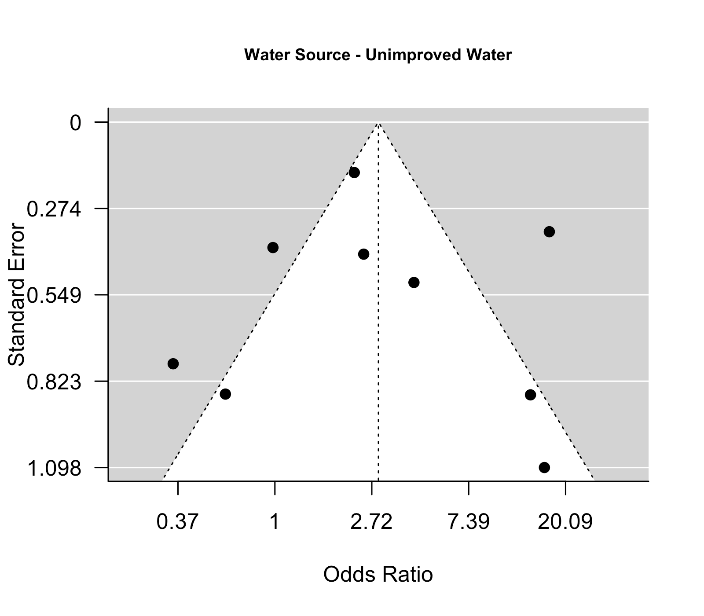

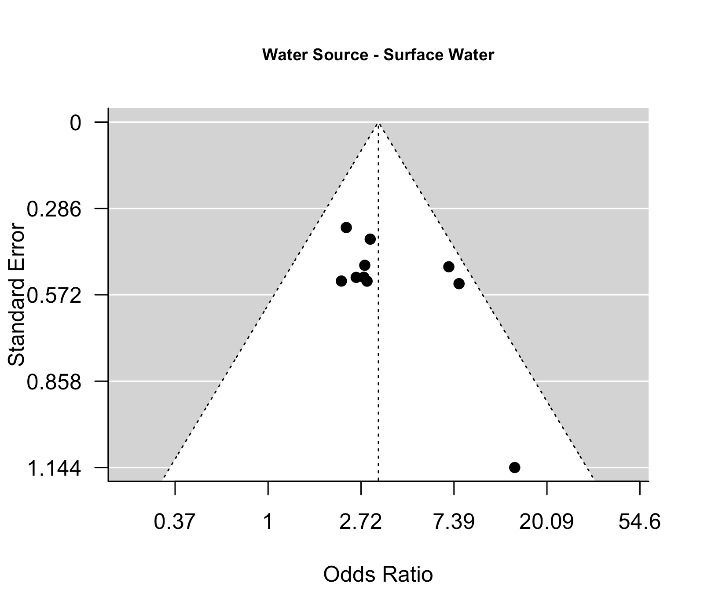


Figure S6. Funnel plot of the untreated (A) and treated water (B).

(A) (B)


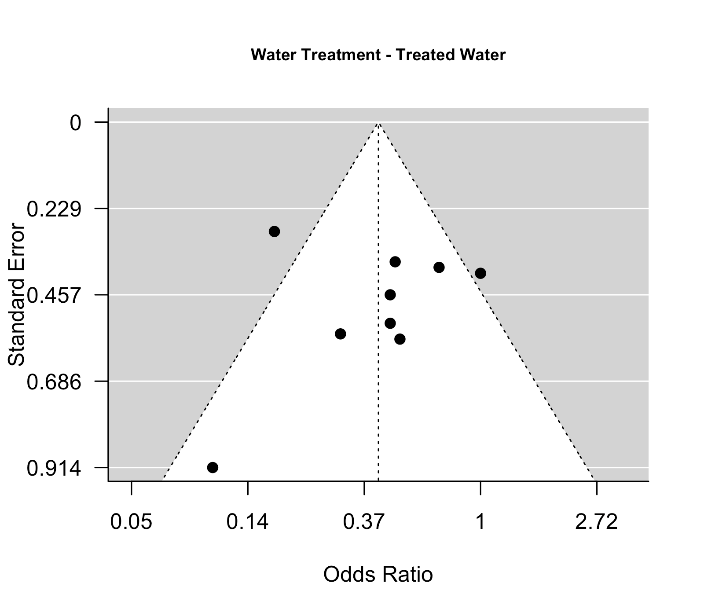

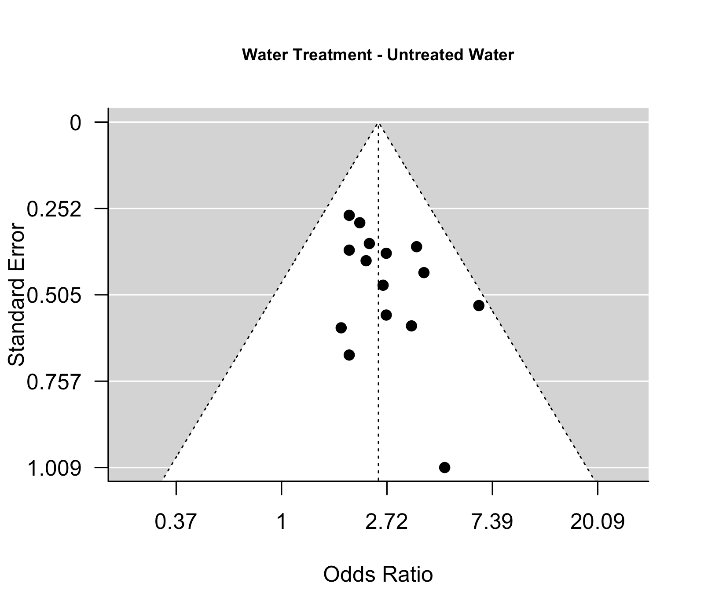


Figure S7. Funnel plot of the treated water. (A), (B), and (C) represent boiled water, chlorinated water, and observation of treatment materials, respectively.

(A) (B)


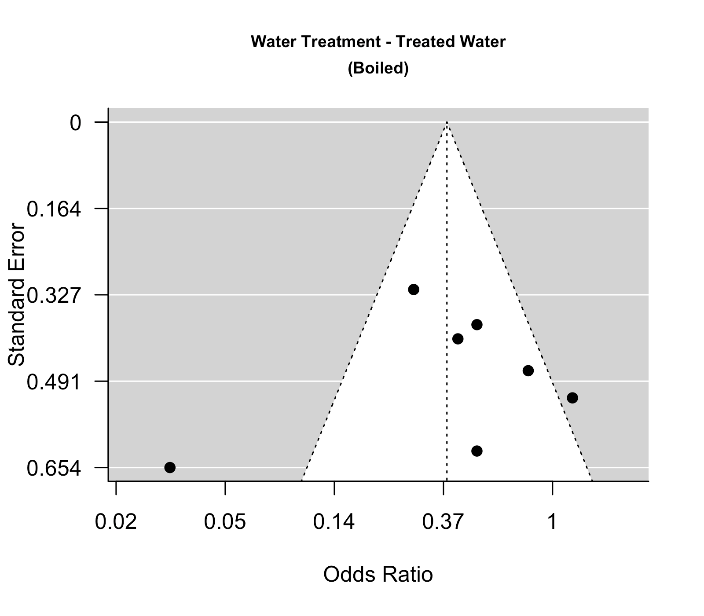

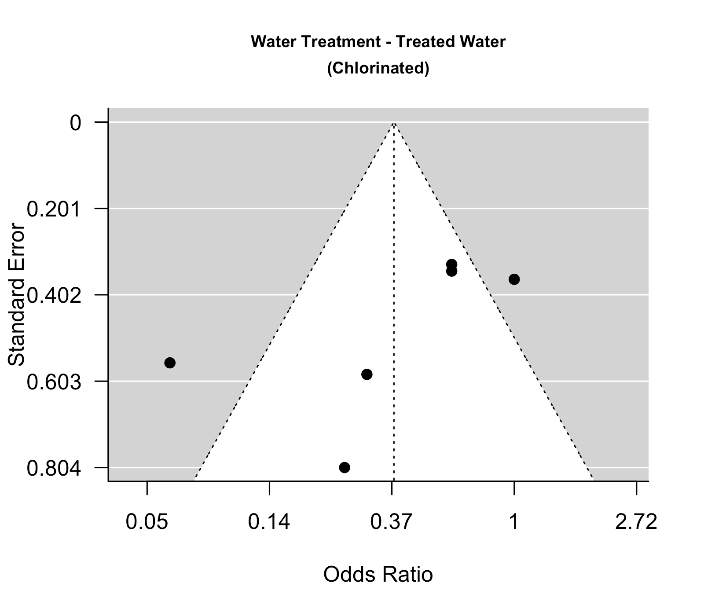


(C)


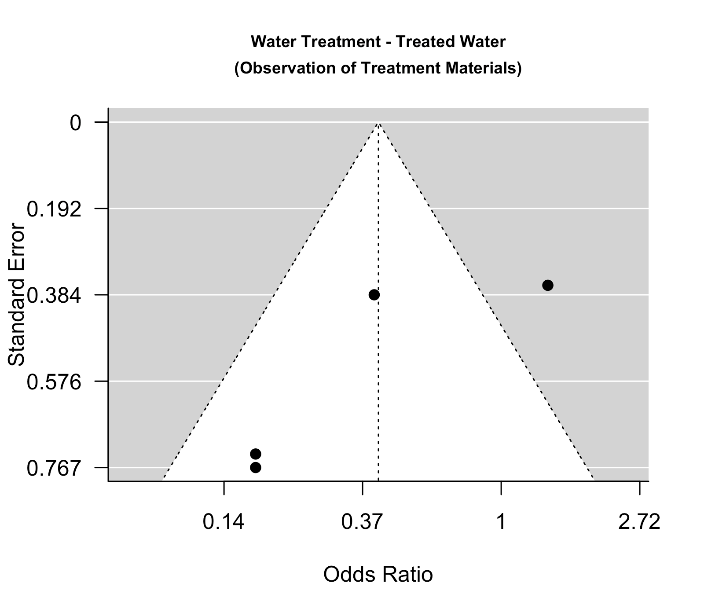


Figure S8. Funnel plot of safe water storage

(A) (B)


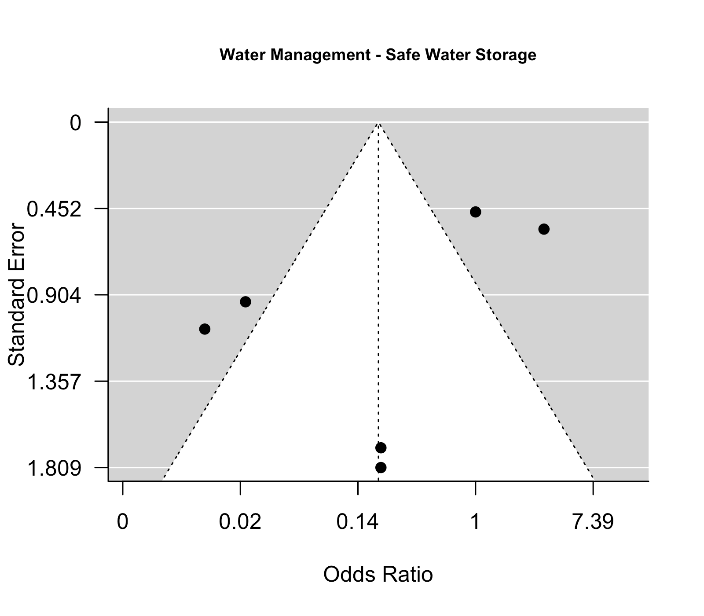

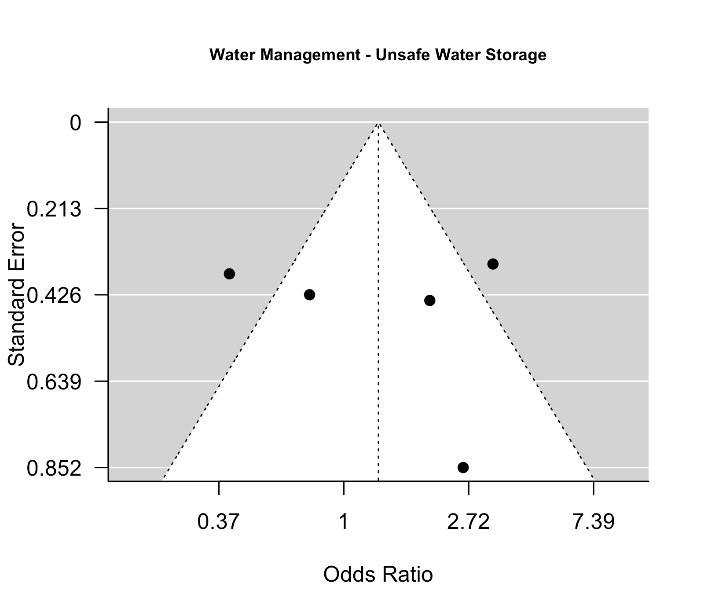


Figure S9. Meta-analyses of the limited water source.


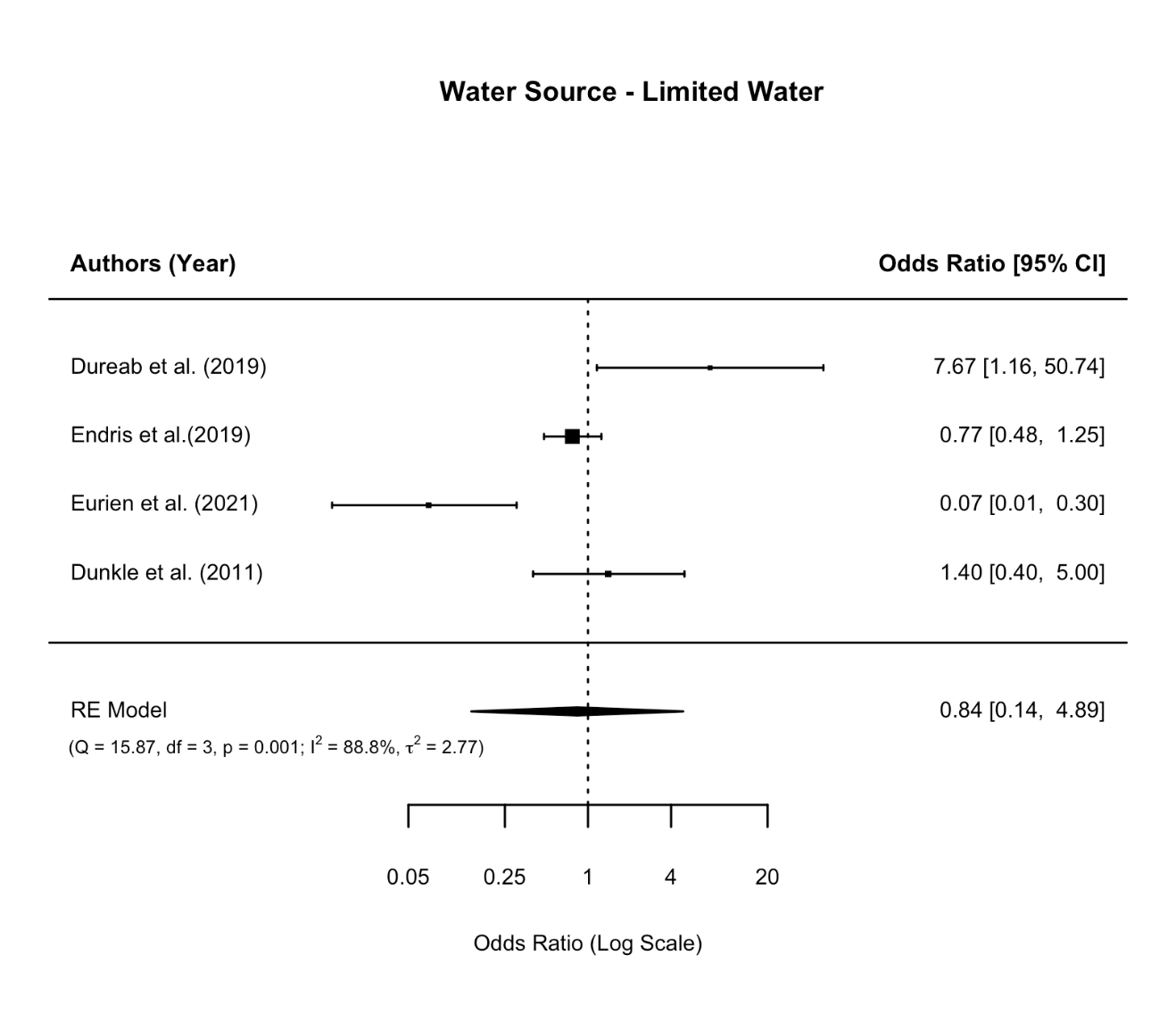


Figure S10. Meta-analyses of treated water (A) and untreated water (B).

(A)


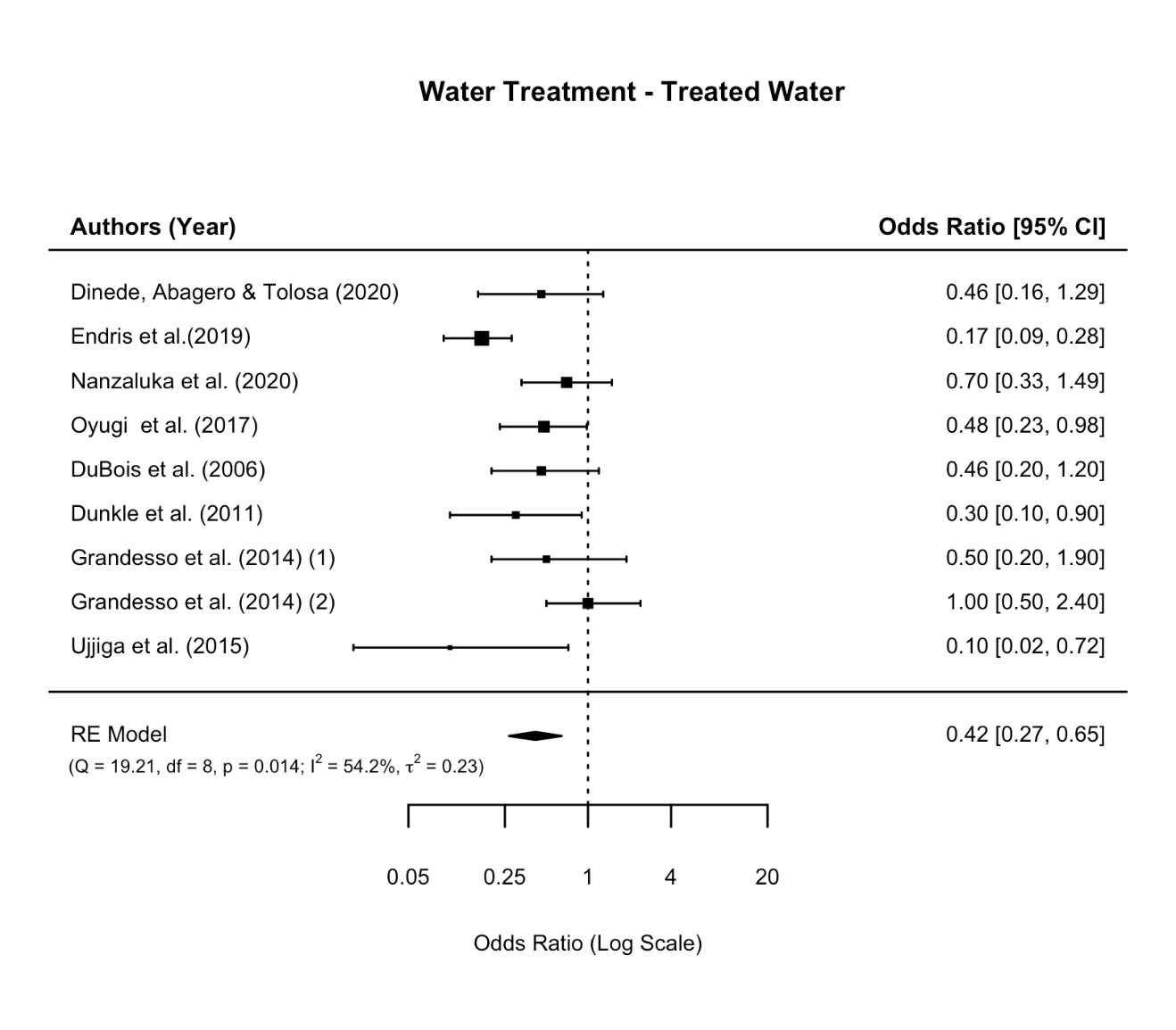


(B)


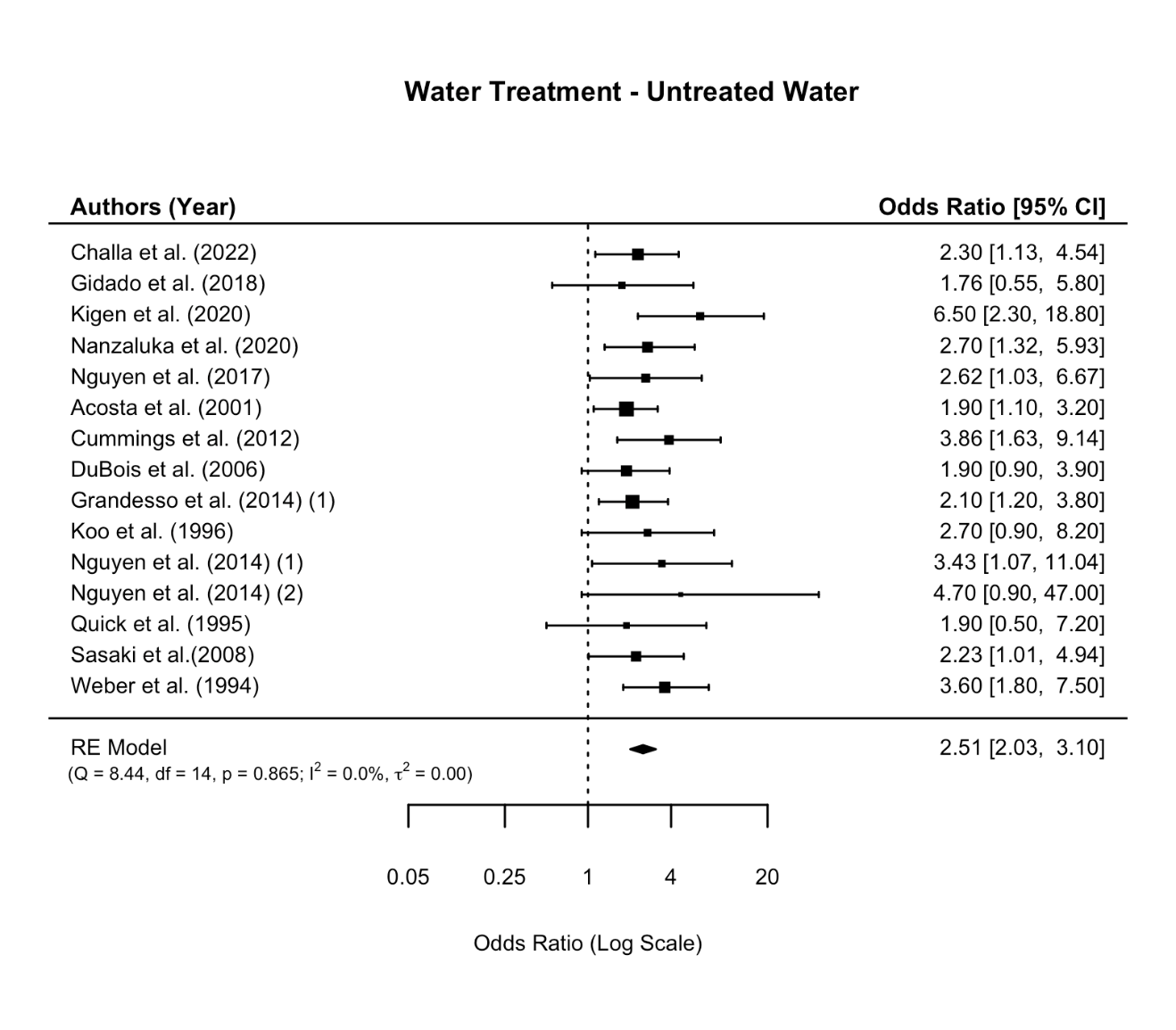


Figure S11. Meta-analyses of safe water storage (A) and unsafe water storage (B)

(A)


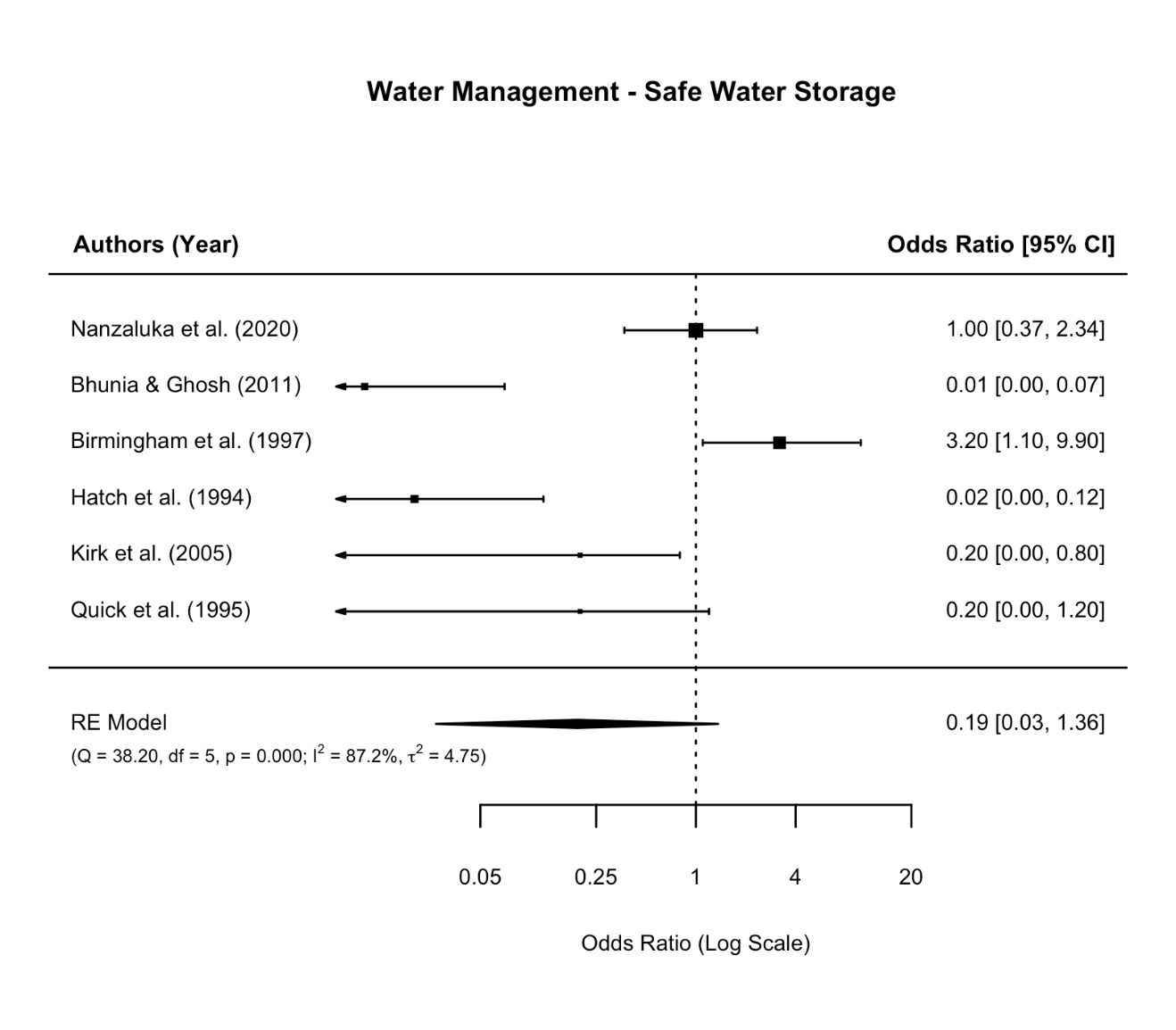


(B)


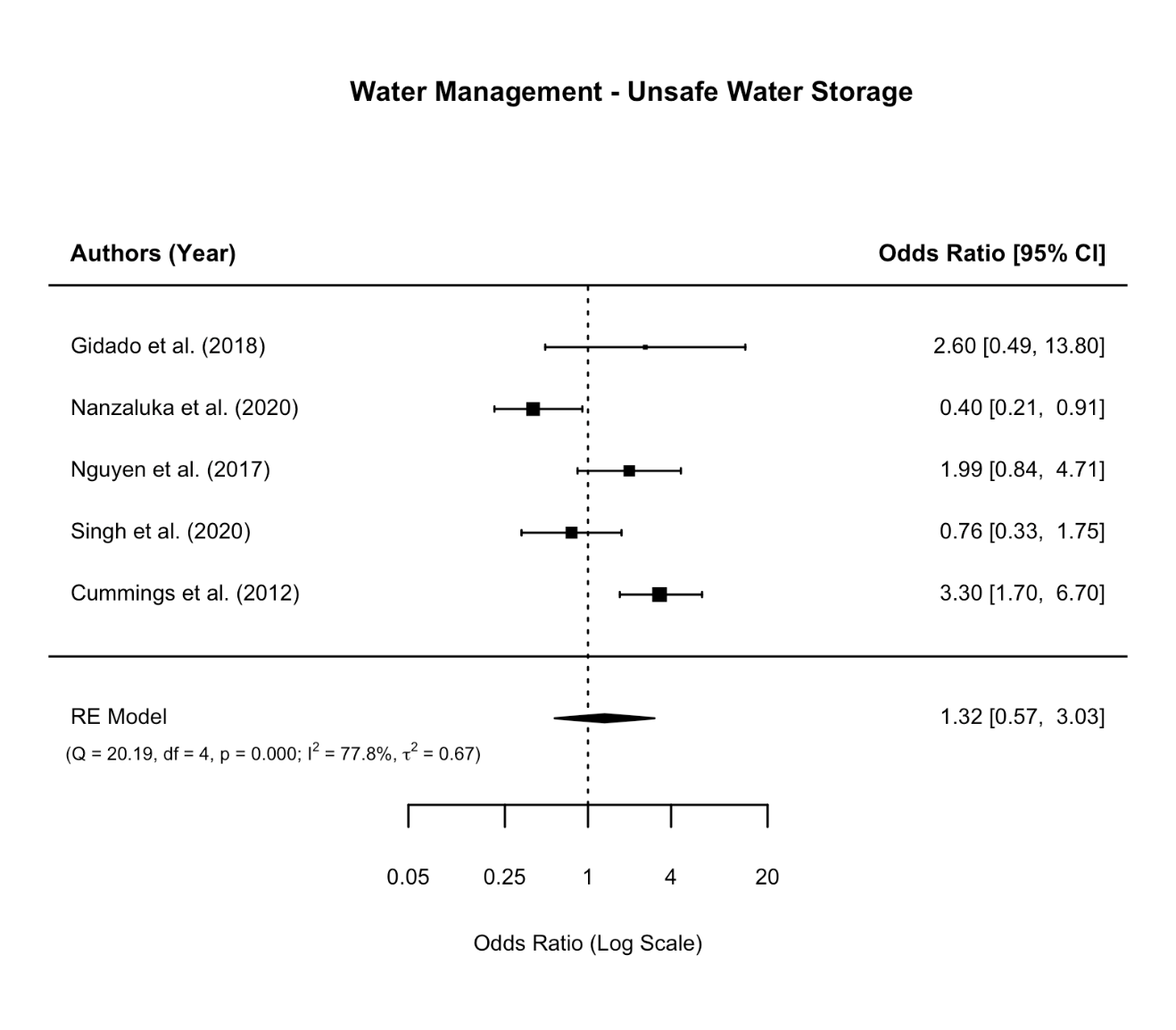


Figure S12. Meta-analysis of the associations between unimproved water and cholera as well as surface water and cholera.

(A)
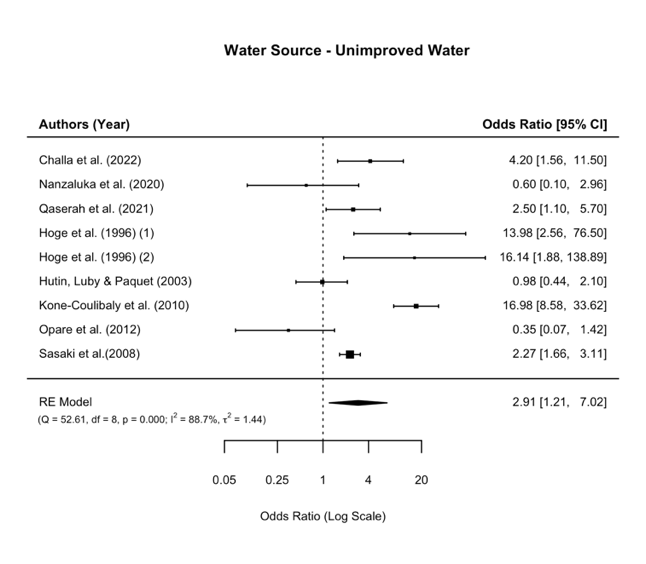


(B)
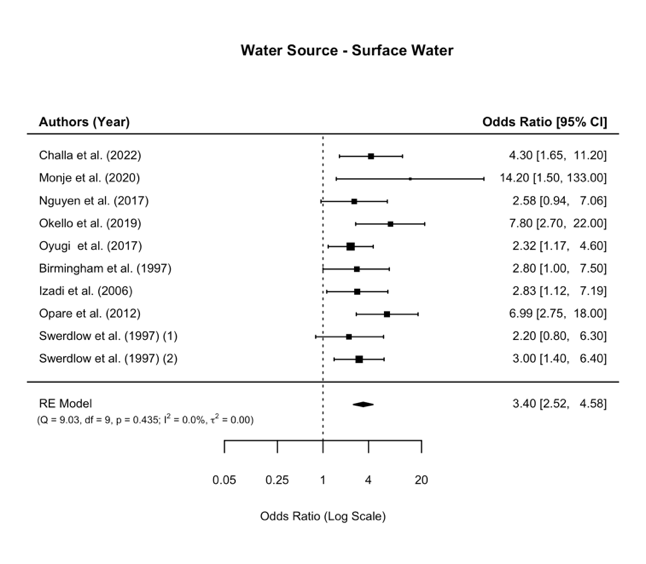


Figure S13. Meta-analysis of the associations between water treatment and cholera.

(A)**
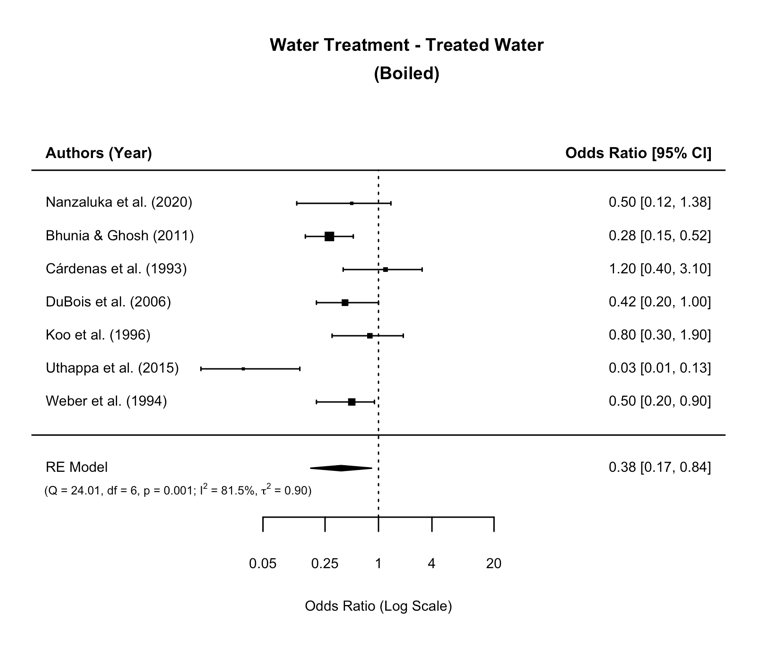
**

(B)**
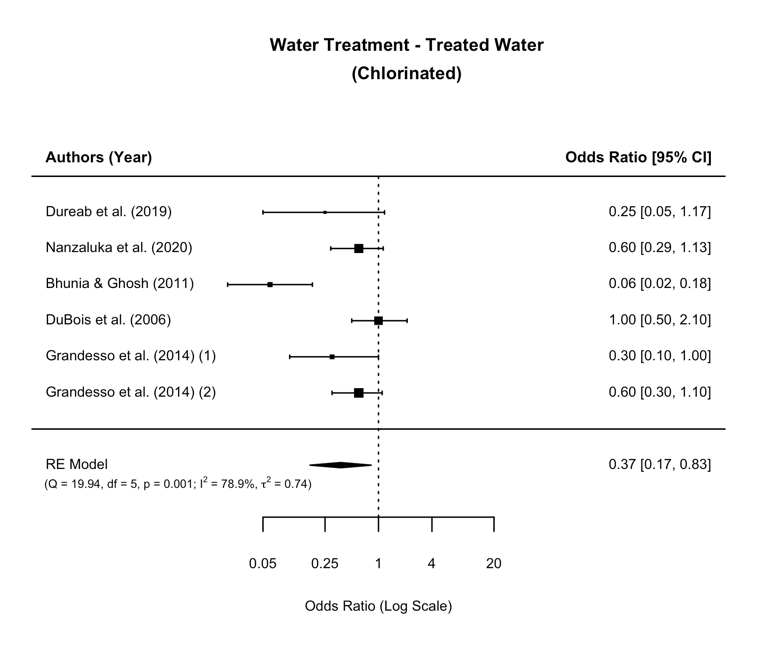
**

(C)**
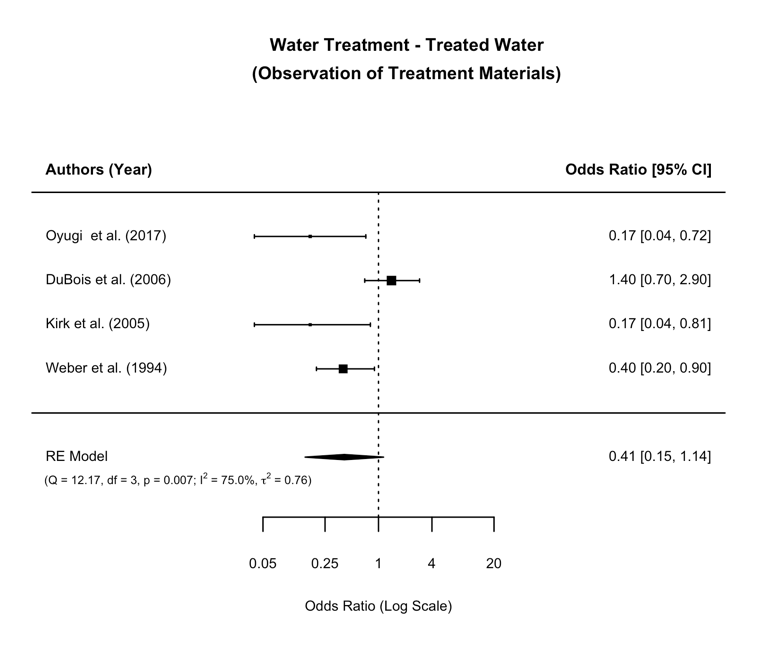
**
