# Supplementary material for: Associations of water quality with cholera in case-control studies: a systematic review and meta-analysis
Source: BMC Infect Dis. 2025 Sep 26;25:1165. doi: 10.1186/s12879-025-11533-x (PMC12465603; doi:10.1186/s12879-025-11533-x)
Supplement: Supplementary file 2 — Supplementary Material 2. [file 12879_2025_11533_MOESM2_ESM.docx]

Table S1. Characteristics of included studies

| **JMP WASH Category** | **Study** | **Country** | **Exposures** | **Effect Size**  **(Odds Ratio)** | **Matched or Adjusted Variables** | **Diagnostic Methods** |
| --- | --- | --- | --- | --- | --- | --- |
| Water Source – Safely Managed  (Bottled Water) | Dunkle et al. (2011) | Haiti | Purchased bottles/filter | 0.9 (0.4–2.3) ***†** | age (matched), sex (matched), neighborhood (matched) | Case definition |
|  | Endris et al. (2019) | Ethiopia | Drinking bottled water | 0.45 (0.15–1.37) **†** | comparable age, comparable occupation | Stool culture |
|  | Hutin, Luby & Paquet (2003) | Nigeria | Drinking bottled water | 0.1 (0.1–0.5) ****†** | age | Case definition |
|  | Koo et al. (1996) | Guatemala | Bottled water | 0.5 (0.2–1.4) ***†** | age (matched), sex (matched), neighborhood (matched) | Stool culture, rectal swab |
|  | Nguyen et al. (2017) | Vietnam | Drinks bottled water | 1.78 (0.81–3.9) **†** | commune (matched), sex (matched), age (matched) | Rectal swab |
| Water Source – Safely Managed  (Sachet Water) | Dunkle et al. (2011) | Haiti | Purchased bags (sachets) | 2.6 (0.7–10.2) ***†** | age (matched), sex (matched), neighborhood (matched) | Case definition |
|  | Issahaku et al. (2020) | Ghana | Drinking sachet water | 11.01 (3.68–32.9) * | drinking pipe-borne water, wash hands before eating | Stool culture, rectal swab |
|  | Mbala-Kingebeni et al. (2021) | Congo | Sachet water consumption: often | 4 (1.6–9.9) ** | age (matched), residence (matched), religion, contact with diarrheal patient since start of epidemic, level of education, procedure before food consumption, procedure before fruit consumption, unprotected source of drinking water of the household, sachet water consumption: rarely, sachet water consumption: very often | Rapid test |
|  | Mbala-Kingebeni et al. (2021) | Congo | Sachet water consumption: very often | 4.1 (1–16.7) ** | age (matched), residence (matched), religion, contact with diarrheal patient since start of epidemic, level of education, procedure before food consumption, procedure before fruit consumption, unprotected source of drinking water of the household, sachet water consumption: rarely, sachet water consumption: often | Rapid test |
|  | Opare et al. (2012) | Ghana | Sachet water | 0.09 (0–0.68) **†** |  | Stool culture |
|  | Qaserah et al. (2021) | Yemen | Bought water or water products in sachets from the street in the last 7 days | 1.7 (1.2–2.8) **†** |  | Case definition |
| Water Source – Safely Managed  (Tap Water) | Acosta et al. (2001) | Tanzania | Water exposure - Inside tap water | 3 (1.7–5) *+ | age (matched), sex (matched) | Stool culture, rectal swab |
|  | Birmingham et al. (1997) | Burundi | Had access to municipal water tap | 0.4 (–) * | sex (matched), age (matched), residence (matched) | Stool culture |
|  | Cárdenas et al. (1993) | Colombia | Drinking municipal water | 7.2 (1.6–32.2) * | Age (matched), gender (matched) | Stool culture |
|  | De Guzman et al. (2015) | Philippines | Drinking from piped water system | 0.22 (–) | age (matched), sex (matched) | Rectal swab |
|  | Dunkle et al. 2011 | Haiti | Piped (house, yard, public tap) | 0.6 (0.2 – 1.6) ***†** | age (matched), sex (matched), neighborhood (matched), | Case definition |
|  | Dureab et al. (2019) | Yemen | Drinking from indoor municipal tap water | 1.5 (0.47–4.84) ****†** | sex (matched), residence (matched), History of contact with cholera patient, drinking from common-source municipal tap water, Using chlorine in the household | Stool culture |
|  | Endris et al. (2019) | Ethiopia | Drinking HH Tap-Water | 0.85 (0.52–1.39) **†** | comparable age, comparable occupation | Stool culture |
|  | Hoge et al. (1996) (1) | Thailand | Consuming untreated tap water | 3.6 (1.69–7.67) ****†** | sex (matched), age (matched), consuming untreated well water, washes hands before eating | Stool culture, rectal swab |
|  | Hoge et al. (1996) (2) | Thailand | Consuming untreated tap water | 2.23 (1.03–4.8) ****†** | sex (matched), age (matched), education (completed primary school), history of contact with person who had diarrhea within household, attendance at gathering, consuming untreated well water, consuming ice, washes hands before eating | Stool culture, rectal swab |
|  | Hutin, Luby & Paquet (2003) | Nigeria | Drinking tap water at home | 0.2 (0.1–0.7) ****†** | age | Case definition |
|  | Issahaku et al. (2020)) | Ghana | Drinking pipe-borne water | 12.13 (1.45–101.32) ** | drinking sachet water, wash hands before eating | Stool culture, rectal swab |
|  | Kone-Coulibaly et al. (2010) | Zimbabwe | Drank tap water | 0.4 (0.02–0.11) ***†** | education | Case definition |
|  | Nanzaluka et al. (2020) | Zambia | Municipal water | 0.9 (0.32–2.57) **†** | age (matched), residence (matched) | Case definition |
|  | Nguyen et al. (2017) | Vietnam | Drinks indoor tap water | 0.89 (0.19–4.11) **†** | commune (matched), sex (matched), age (matched) | Rectal swab |
|  | Reller et al. (2001) | Madagascar | Water from home faucet | 0.1 (0 – 0.9) | age (matched), sex (matched), neighborhood (matched) | Stool culture |
|  | Rodrigues, Brun & Sandstrom (1997) | Guinea-Bissau | Tap water | 11.7 (3.6–37.9) | age (matched), gender (matched), residence (matched) | Case definition |
|  | Singh et al. (2020) | India | Drinking water - Untreated municipal water | 2.38** (1.01–6.22) | age ≤25 y, religion, no knowledge regarding diarrhea transmission, poor hand hygiene | Stool culture |
|  | St Louis et al. (1990) | Guinea | Drinking piped water | 1.36 (0.35–5.17) | age (matched), sex (matched) | Stool culture, rectal swab |
|  | von Seidlein et al. (2008) | Mozambique | With access to tap water at home | 3.3 (0.94–11.6) ****†** | neighborhood (matched), age, sex | Stool culture, rectal swab |
| Water Source - Basic | Beatty et al. (2004) (1) | Marshall Islands | Rainwater | 0.08 (0–0.69) * | sex (matched), age (matched), neighborhood (matched) | Case definition |
|  | Beatty et al. (2004) (2) | Marshall Islands | Rainwater | 0.01 (0 – 1) * | sex (matched), age (matched), neighborhood (matched) | Serologic test |
|  | Bhunia & Ghosh (2011) | India | Tube well water | 0.51 (0.26–1) ***†** | residence (matched), race (matched), SES (matched) | Stool culture |
|  | Kirk et al. (2005) | Micronesia | Untreated rainwater main source | 8 (1.1–355) **†** | age (matched), sex (matched) | Case definition |
|  | Nanzaluka et al. (2020) | Zambia | Borehole | 2.4 (1.1–5.6) ** | age (matched), residence (matched), Had close contact with a cholera case, sex | Case definition |
|  | Nguyen et al. (2017) | Vietnam | Drinks stored rainwater | 0.17 (0.04 – 0.63) ****†** | commune (matched), sex (matched), age (matched), primary education or illiterate, lives with people who had acute diarrhea, main source of water close to a toilet, self-perceived changes in the color, odor, appearance and taste of water, bathes with sedimented river water, drinks boiled water: Sometimes, often or never, drinks bottled water, drinks indoor tap water | Rectal swab |
|  | Okello et al. (2019) | Uganda | Untreated borehole water for drinking | 0.31 (0.13–0.65) ***†** | age (matched), residence (matched) | Stool culture |
|  | Opare et al. (2012) | Ghana | Borehole | 0.86 (0.2–3.47) |  | Stool culture |
|  | Shapiro et al. (1999) | Kenya | Drinking water from a borehole | 0.3 (0.1–0.8) * | age (matched), sex (matched), clinic (matched) | Stool culture, rectal swab |
|  | Shapiro et al. (1999) | Kenya | Drinking rainwater | 0.4 (0.2–0.8) * | age (matched), sex (matched), clinic (matched) | Stool culture, rectal swab |
| Water Source - Limited | Dunkle et al. (2011) | Haiti | Tanker | 1.4 (0.4–5) ***†** | age (matched), sex (matched), neighborhood (matched) | Case definition |
|  | Dunkle et al. (2011) | Haiti | Bladder | 0 (0–0.9) * | age (matched), sex (matched), neighborhood (matched) | Case definition |
|  | Dureab et al. (2019) | Yemen | Drinking from common-source municipal tap water | 7.67 (1.16–50.74) ****†** | sex (matched), residence (matched), history of contact with cholera patient, drinking from indoor municipal tap water, using chlorine in the household | Stool culture |
|  | Dutta et al. (2021) | India | Tube wells | 1 (0.5–2.2) |  | Stool culture |
|  | Endris et al. (2019) | Ethiopia | Drinking Tanker Water (Roto) | 2.01 (0.12–32.48) | comparable age, comparable occupation | Stool culture |
|  | Endris et al. (2019) | Ethiopia | Drinking Communal Tap-water | 0.77 (0.48–1.25) **†** | comparable age, comparable occupation | Stool culture |
|  | Eurien et al. (2021) | Uganda | Drank water from Well B only (were privately-owned and protected) | 0.83 (0.11–6.6) * | age (matched) | Stool culture |
|  | Eurien et al. (2021) | Uganda | Drank water from token tap only | 0.07 (0.014 – 0.304) ***†** | age (matched) | Stool culture |
|  | Eurien et al. (2021) | Uganda | Drank water from Well A only (were privately-owned and protected) | 0 (0–undefined) * | age (matched) | Stool culture |
|  | Fredrick et al. (2015) | India | Consumption of water from the public drinking-water system | 37 (4–285) | gender (matched), age (matched), neighborhood (matched) | Rectal swap |
|  | Monje et al. (2020) | Uganda | Drank Tank only | 11.6 (1.4–94) | Regular washing of hands before food and after defecation | Rapid test |
|  | Mridha et al. (2011) | India | Reservoir within mill compound as the main source of drinking-water during work | 12.1 (4.3–34) ** | regular washing of hands before food and after defecation | Rectal swab |
|  | Siddiqui et al. (2006) | Pakistan | Reservoir water | 7.1 (3–16.9) | age (matched), sex (matched), neighborhood (matched) | Stool culture |
|  | Uthappa et al. (2015) | India | Drinking exclusive Overhead tank water | 31.94 (7.31–139.53) ** | age (matched), residence (matched), SES, presence of house flies, mean household members (4), regularly drink boiled water, wash hands before eating | Rectal swab |
| Water Source – Unimproved | Challa et al. (2022) | Ethiopia | Hand dug well | 3.6 (1.3 – 10.2) ** | residence, SES, Spring, deep well, river, no use water purifying, Have no toilet at household, sometimes handwashing after | Case definition |
|  | Challa et al. (2022) | Ethiopia | Deep well | 4.2 (1.56 – 11.5) ****†** | residence, SES, spring, hand dug well, river, no use water purifying, have no toilet at household, sometimes handwashing after | Case definition |
|  | Challa et al. (2022) | Ethiopia | Spring | 2.9 (1.04–8.2) ****†** | residence, SES, hand dug well, deep well, river, no use water purifying, have no toilet at household, sometimes handwashing after | Case definition |
|  | De Guzman et al. (2015) | Philippines | Drinking from free-flow wells | 3.62 (–) | age (matched), sex (matched) | Rectal swab |
|  | De Guzman et al. (2015) | Philippines | Drinking from open dug wells | 2.81 (–) | age (matched), sex (matched) | Rectal swab |
|  | Dutta et al. (2021) | India | Paddy field dug wells | 4 (1.9–8.1) |  | Stool culture |
|  | Dutta et al. (2021) | India | Village dug wells | 1 (0.5–2.2) |  | Stool culture |
|  | Eurien et al. (2021) | Uganda | Drank water from Well C only | 21 (4.6–93) * | age (matched) | Stool culture |
|  | Eurien et al. (2021) | Uganda | Drank water both from token tap &Well C | 0.7 (0.18–2.7) * | age (matched) | Stool culture |
|  | Eurien et al. (2021) | Uganda | Drank water from both tokens tap & Well B | 1 (0.12–9.1) * | age (matched) | Stool culture |
|  | Eurien et al. (2021) | Uganda | Drank water from both token tap & Well A | 0 (0–undefined) * | age (matched) | Stool culture |
|  | Hoge et al. (1996) (1) | Thailand | Consuming untreated well water | 13.98 (2.56 – 76.5) ****†** | sex (matched), age (matched), consuming untreated tap water, washes hands before eating gathering | Stool culture, rectal swab |
|  | Hoge et al. (1996) (2) | Thailand | Consuming untreated well water | 16.14 (1.88 – 138.89) ****†** | sex (matched), age (matched), education (completed primary school), history of contact with person who had diarrhea within household, attendance at gathering, consuming untreated tap water, consuming ice, washes hands before eating | Stool culture, rectal swab |
|  | Hutin, Luby & Paquet (2003) | Nigeria | Drinking water from a well | 0.98 (0.44–2.1) ****†** | Age | Case definition |
|  | Izadi et al. (2005) | Iran | Deep well | 1.87 (0.39–0.88) |  | Stool culture |
|  | Izadi et al. (2005) | Iran | Aqueducts | 1.76 (0.37–8.39) |  | Stool culture |
|  | Kone-Coulibaly et al. (2010) | Zimbabwe | Drank unprotected well water | 16.98 (8.58–33.62) **†** |  | Case definition |
|  | Monje et al. (2020) | Uganda | Drank Spring only | 0 (–) |  | Rapid test |
|  | Monje et al. (2020) | Uganda | Drank Spring and tank only | 0 (–) |  | Rapid test |
|  | Moren et al. (1991) | Malawi | Shallow wells | 4.5 (1–20.8) * | age (matched), sex (matched) | Stool culture |
|  | Nanzaluka et al. (2020) | Zambia | Shallow well | 0.6 (0.1–2.96) **†** | age (matched), residence (matched) | Case definition |
|  | Nsagha et al. (2015) | Cameroon | Spring as drinking water source | 0.58 (0.07–5.16) | neighborhood (matched) | Case definition |
|  | O'Connor et al. (2011) | Haiti | Well | 0.3 (0.1–2.5) * | age (matched), sex (matched) | Case definition |
|  | Opare et al. (2012) | Ghana | Well | 0.35 (0.07 – 1.41) **†** |  | Stool culture |
|  | Qaserah et al. (2021) | Yemen | Public well | 2.5 (1.1 – 5.7) ****†** | residence (matched), Storing water in containers, public toilet | Case definition |
|  | Rodrigues, Brun & Sandstrom (1997) | Guinea-Bissau | Well | 13 (4–42.1) | age (matched), gender (matched), residence (matched) | Case definition |
|  | Sasaki et al. (2008) | Zambia | Drink water from a shallow well | 2.269 (1.657 – 3.107) ****†** | sex (matched), age (matched), average monthly income, number of family members, households without latrine, households without drainage | Stool culture, rectal swab |
| Water Source – Surface Water | Birmingham et al. (1997) | Burundi | Drank lake water | 2.8 (1–7.5) ****†** | sex (matched), age (matched), residence (matched), bathed in lake, used 20 L jerry-can store drinking water | Stool culture |
|  | Birmingham et al. (1997) | Burundi | Drank river water | 2.5 (–) * | sex (matched), age (matched), residence (matched) | Stool culture |
|  | Challa et al. (2022) | Ethiopia | River | 4.3 (1.65–11.2) ****†** | residence, SES, spring, hand dug well, deep well, no use water purifying, have no toilet at household, sometimes handwashing after | Case definition |
|  | Dan-Nwafor et al. (2019) | Nigeria | River Zamani | 14.2 (5.5–36.8) |  | Rapid test |
|  | Dutta et al. (2021) | India | Puddles | 1.12 (0.4–3.6) |  | Stool culture |
|  | Izadi et al. (2006) | Iran | Well, river, aqueducts, tankers | 2.83 (1.12–7.19) ***†** | residence (matched), Illiteracy, sex | Rectal swab |
|  | Kwesiga et al. (2017) | Uganda | Source of drinking water: River or Stream | 1.3 (0.57–2.8) | age (matched), residence (matched) | Stool culture |
|  | Monje et al. (2020) | Uganda | Drank Stream | 14.2 (1.5–133) **†** |  | Rapid test |
|  | Monje et al. (2020) | Uganda | Drank Spring and stream | 0 (–) |  | Rapid test |
|  | Monje et al. (2020) | Uganda | Drank Tank and stream only | 17.3 (2.2–137) |  | Rapid test |
|  | Monje et al. (2020) | Uganda | Drank Stream and tank and spring | ∞ (–) |  | Rapid test |
|  | Nguyen et al. (2017) | Vietnam | Drinks sedimented river water | 2.58 (0.94–7.06) **†** | commune (matched), sex (matched), age (matched), main source of water close to a toilet, Self-perceived changes in the color, odor, appearance and taste of water, bathes with sedimented river water, drinks boiled water: sometimes, often or never, drinks bottled water, drinks indoor tap water | Rectal swab |
|  | Oguttu et al. (2017) | Uganda | Lake Albert Site C | 6.7 ( 2.5–17) * |  | Stool culture |
|  | Oguttu et al. (2017) | Uganda | Lake Albert Site B | 1.8 (0.64–5.3) * |  | Stool culture |
|  | Okello et al. (2019) | Uganda | Untreated Cheptui river water for drinking | 7.8 (2.7–22) ***†** | age (matched), residence (matched) | Stool culture |
|  | Okello et al. (2019) | Uganda | Untreated swamp water for drinking | 2.5 (0.8–8) * | age (matched), residence (matched) | Stool culture |
|  | Opare et al. (2012) | Ghana | Stream/river | 6.99 (2.75–18) **†** |  | Stool culture |
|  | Oyugi et al. (2017) | Kenya | Riana River as drinking source | 2.32 (1.17–4.6) ***†** | age (matched), residence (matched) | Stool culture |
|  | Pande et al. (2018) | Uganda | Lakeshore water | 16 (2.4–107) * | age (matched) | Stool culture |
|  | Rosewell et al. (2012) | Papua New Guinea | River as drinking water | 2.5 (1.2–5.2) |  | Stool culture, rapid test |
|  | Shapiro et al. (1999) | Kenya | Drinking water from Lake Victoria | 6.5 (1.6–25.5) * | age (matched), sex (matched), clinic (matched) | Stool culture,  rectal swab |
|  | Shapiro et al. (1999) | Kenya | Drinking water from a stream | 10.8 (1.7–70.1) * | age (matched), sex (matched), clinic (matched) | Stool culture,  rectal swab |
|  | Shultz al. (2009) | Kenya | Drinking river water | 1.6 (0.4–5.8) * | age (matched), residence (matched) | Case definition |
|  | Swerdlow et al. (1997) (1) | Malawi | Drank any river water | 2.2 (0.8–6.3) ***†** | age (matched), sex (matched), date of arrival (matched) | Rectal swab |
|  | Swerdlow et al. (1997) (2) | Malawi | Drank any river water | 3 (1.4–6.4) ***†** |  | Rectal swab |
|  | Swerdlow et al. (1997) (2) | Malawi | Among persons who went to river - Drank river water | 16.1 (2–351.2) * |  | Rectal swab |
| Water Treatment – Treated Water | Beatty et al. (2004) (1) | Marshall Islands | Use of powdered drink mix for treatment | 0.18 (0.08–0.37) * | sex (matched), age (matched), neighborhood (matched) | Case definition |
|  | Beatty et al. (2004) (2) | Marshall Islands | Use of powdered drink mix for treatment | 0.18 (0.06 – 0.44) * | sex (matched), age (matched), neighborhood (matched) | Serologic test |
|  | Dinede, Abagero & Tolosa (2020) | Ethiopia | Treating drinking water with chemical | 0.46 (0.16–1.29) **†** |  | Stool culture |
|  | DuBois et al. (2006) | Zambia | Regularly use any water treatment | 0.46 (0.2 – 1.2) ***†** | sex (matched), age (matched), residence (matched) | Rectal swab |
|  | Dunkle et al. (2011) | Haiti | Boiling water or using a chlorine product <= 3d before illness | 0.5 (0.2–1.2) * | age (matched), sex (matched), neighborhood (matched) | Case definition |
|  | Dunkle et al. (2011) | Haiti | Boiling water or using a chlorine product before November 1, 2010 | 0.3 (0.1–0.9) *+ | age (matched), sex (matched), neighborhood (matched) | Case definition |
|  | Endris et al. (2019) | Ethiopia | Treat water before drinking | 0.17 (0.09–0.28) **†** | comparable age, comparable occupation | Stool culture |
|  | Grandesso et al. (2014) (1) | Haiti | Residual free chlorine in drinking water >0.2 mg/l | 0.5 (0.2–1.9) ****†** | sex (matched), age (matched), SES, presence of E. coli in drinking water stored at home, receiving information on cholera prevention via television, receiving information on cholera prevention in training session, number of member in household, sharing latrine with someone suffering from diarrhoea, always chlorinate water before drinking | Rapid test |
|  | Grandesso et al. (2014) (2) | Haiti | Residual free chlorine in drinking water >0.2 mg/l | 1 (0.5–2.4) ****†** | sex (matched), age (matched), SES, presence of E. coli in drinking water stored at home, receiving information on cholera prevention via television, receiving information on cholera prevention at church, using untreated water for washing, sharing latrine with someone suffering from diarrhoea | Rapid test |
|  | Mahamud et al. (2012) | Kenya | Treated water before drinking | 0.25 (0.07–0.89) * | age (matched), residence (matched) | Case definition |
|  | Nanzaluka et al. (2020) | Zambia | Reported treating water | 0.7 (0.33–1.49) **†** | age (matched), residence (matched) | Case definition |
|  | O'Connor et al. (2011) | Haiti | Treating drinking water before the outbreak | 0.9 (0.4–2.3) * | age (matched), sex (matched) | Case definition |
|  | O'Connor et al. (2011) | Haiti | Treating drinking water 3d before illness onset (during outbreak) | 0.2 (0.1–0.7) * | age (matched), sex (matched) | Case definition |
|  | Oyugi et al. (2017) | Kenya | Treating drinking water | 0.48 (0.23–0.98) **†** | age (matched), residence (matched) | Stool culture |
|  | Ujjiga et al. (2015) | South Sudan | Treated drinking water at home | 0.1 (0.02–0.72) ****†** | age (matched), sex (matched), neighborhood (matched), SES, had 2 oral vaccine doses, Traveled outside home village before onset of illness | Stool culture, rapid test |
| Water Treatment – Treated Water  (Boiled) | Beatty et al. (2004) (1) | Marshall Islands | Boiling | 0.47 (0.22–0.98) | sex (matched), age (matched), neighborhood (matched) | Case definition |
|  | Beatty et al. (2004) (2) | Marshall Islands | Boiling | 0.44 (0.17–1.12) | sex (matched), age (matched), neighborhood (matched) | Serologic test |
|  | Bhunia & Ghosh (2011) | India | Boiled water | 0.28 (0.15–0.52) ***†** | residence (matched), race (matched), SES (matched) | Stool culture |
|  | Cárdenas et al. (1993) | Colombia | Drinking water boiled | 1.2 (0.4–3.1) **†** | age, gender | Stool culture |
|  | De Guzman et al. (2015) | Philippines | Boiling water | 0.88 (–) | age (matched), sex (matched) | Rectal swab |
|  | DuBois et al. (2006) | Zambia | Regularly boil drinking water | 0.42 (0.2–1) ***†** | sex (matched), age (matched), residence (matched) | Rectal swab |
|  | Koo et al. (1996) | Guatemala | Always boil | 0.8 (0.3–1.9) ***†** | age (matched), sex (matched), neighborhood (matched) | Stool culture, rectal swab |
|  | Nanzaluka et al. (2020) | Zambia | Reported treating water by boiling | 0.5 (0.12–1.38) **†** | age (matched), residence (matched) | Case definition |
|  | Ries et al. (1992) (1) | Peru | Always drink boiled water | 0.3 (0.1–0.6) * | age (matched), sex (matched) | Rectal swab |
|  | Ries et al. (1992) (2) | Peru | Always drink boiled water | 0.1 (0.02–0.5) * | age (matched), sex (matched) | Rectal swab, blood culture |
|  | Uthappa et al. (2015) | India | Regularly drink boiled water | 0.03 (0.01–0.13) ****†** | age (matched), residence (matched), SES, presence of house flies, mean household members (4), drinking exclusive vverhead tank water, wash hands before eating | Rectal swab |
|  | Weber et al. (1994) | Ecuador | Always boiling drinking water at home | 0.5 (0.2–0.9) **†** | age (matched), sex (matched), neighborhood (matched) | Rectal swab |
|  | Weber et al. (1994) | Ecuador | Always boiling water for refreshments at home | 0.4 (0.1–1.4) | age (matched), sex (matched), neighborhood (matched) | Rectal swab |
| Water Treatment – Treated Water  (Chlorinated) | Bhunia & Ghosh (2011) | India | Chlorine-treated water | 0.06 (0.02–0.18) ***†** | residence (matched), race (matched), SES (matched) | Stool culture |
|  | DuBois et al. (2006) | Zambia | Reported chlorination of stored water | 1 (0.5–2.1) ***†** | sex (matched), age (matched), residence (matched) | Rectal swab |
|  | DuBois et al. (2006) | Zambia | Free chlorine present in stored water | 1.5 (0.7–3.5) * | sex (matched), age (matched), residence (matched) | Rectal swab |
|  | Dureab et al. (2019) | Yemen | Using chlorine in the household | 0.25 (0.05–1.17) ****†** | sex (matched), residence (matched), history of contact with cholera patient, drinking from indoor municipal tap water, drinking from common-source municipal tap water | Stool culture |
|  | Grandesso et al. (2014) (1) | Haiti | Always chlorinate water before drinking (self-reported) | 0.5 (0.3–0.9) ****†** | sex (matched), age (matched), SES, presence of E. coli in drinking water stored at home, receiving information on cholera prevention via television, receiving information on cholera prevention in training session, number of member in household, chlorine level in water, sharing latrine with someone suffering from diarrhoea | Rapid test |
|  | Grandesso et al. (2014) (2) | Haiti | Always chlorinate water before drinking (self-reported) | 0.6 (0.3–1.1) ***†** | sex (matched), age (matched), SES | Rapid test |
|  | Nanzaluka et al. (2020) | Zambia | Reported treating water by chlorination | 1 (0.49–1.99) | age (matched), residence (matched) | Case definition |
|  | Nanzaluka et al. (2020) | Zambia | Reported chlorinating water within 5 days of survey | 0.6 (0.29–1.13) **†** | age (matched), residence (matched) | Case definition |
|  | O'Connor et al. (2011) | Haiti | Residual chlorine presence in home drinkign water >= .1 mg/L | 0.4 (0.1–1.3) * | age (matched), sex (matched) | Case definition |
|  | O'Connor et al. (2011) | Haiti | Residual chlorine presence in home drinking water >= .5 mg/L | 0.4 (0.1–1.8) * | age (matched), sex (matched) | Case definition |
|  | Reller et al. (2001) | Madagascar | Water treated with sodium hypochlorite solution | 0.1 (0–1.2) | age (matched), sex (matched), neighborhood (matched) | Stool culture |
| Water Treatment – Treated Water  (Observation of Treatment Materials) | DuBois et al. (2006) | Zambia | Bottle of chlorine water treatment solution observed | 1.4 (0.7–2.9) **†** | sex (matched), age (matched), residence (matched) | Rectal swab |
|  | Kirk et al. (2005) | Micronesia | Clorox present in house | 0.17 (0.04–0.81) ****†** | sex (matched), age (matched), residence (matched) | Rectal swab |
|  | Nanzaluka et al. (2020) | Zambia | Surveyor observed chlorine in house | 2.4 (1–5.81) | age (matched), residence (matched) | Case definition |
|  | O'Connor et al. (2011) | Haiti | Water treatment product in home | 0.8 (0.3–2.4) * | age (matched), sex (matched) | Case definition |
|  | Oyugi et al. (2017) | Kenya | Having cholerine in household | 0.17 (0.04–0.72) **†** | age (matched), residence (matched) | Stool culture |
|  | Weber et al. (1994) | Ecuador | Boiled water present in home | 0.4 (0.2–0.9) **†** | age (matched), sex (matched), neighborhood (matched) | Rectal swab, blood culture |
| Water Treatment - Untreated | Acosta et al. (2001) | Tanzania | Water exposure - unboiled drinking water | 1.9 (1.1–3.2) ***†** | age (matched), sex (matched) | Stool culture, rectal swab |
|  | Acosta et al. (2001) | Tanzania | Water exposure - unfiltered drinking water | 1.7 (0.9–3) * | age (matched), sex (matched) | Stool culture, rectal swab |
|  | Challa et al. (2022) | Ethiopia | No use water purifying | 2.3 (1.13–4.54) ****†** | residence, SES, spring, hand dug well, deep well, river, have no toilet at household, sometimes handwashing after | Case definition |
|  | Cummings et al. (2012) | Uganda | Does not use chlorine tablets to treat drinking water | 3.86 (1.63–9.14) ****†** | Resides in same household as another cholera case, sex, age group: children, does not use latrine to dispose of children's faeces | Stool culture |
|  | DuBois et al. (2006) | Zambia | Drank any untreated water | 1.9 (0.9–3.9) ***†** | sex (matched), age (matched), residence (matched) | Rectal swab |
|  | Dutta et al. (2021) | India | Did not treat water before drinking | 2.2 (0.7–6.9) |  | Stool culture |
|  | Fredrick et al. (2015) | India | Not boiling water | 35 (4–269) * | gender (matched), age (matched), neighborhood (matched) | Rectal swab |
|  | Gidado et al. (2018) | Nigeria | Not preparing water before drinking | 1.76 (0.55–5.8) **†** |  | Stool culture |
|  | Grandesso et al. (2014) (1) | Haiti | Using untreated water to wash dishes | 2.1 (1.2–3.8) **†** | sex (matched), age (matched), SES | Rapid test |
|  | Kigen et al. (2020) | Kenya | Not treating drinking water last 5 days | 6.5 (2.3–18.8) ****†** | less than 12 years of schooling (secondary), lack of health education on diarrheal illness or on cholera last 6 months | Stool culture |
|  | Koo et al. (1996) | Guatemala | Untreated water | 2.7 (0.9–8.2) ***†** | age (matched), sex (matched), neighborhood (matched) | Stool culture, rectal swab |
|  | Kwesiga et al. (2017) | Uganda | Drinking unboiled/untreated water | 4.8 (1.3–18) * | age (matched), residence (matched) | Stool culture |
|  | Mugoya et al. (2008) (1) | Kenya | Not treating drinking water | 4.9 (1.6–15) ** | age (matched), neighborhood (matched), unsound latrine superstructure,sStoring water in a bucket | Stool culture |
|  | Mugoya et al. (2008) (2) | Kenya | Not treating drinking water | 5 (1.4–18) | age (matched), neighborhood (matched) | Stool culture |
|  | Mujica et al. (1994) | Peru | Unboiled drinking water | 2.9 (1.3–6.43) * | age (matched), sex (matched) | Stool culture |
|  | Nanzaluka et al. (2020) | Zambia | Reported drinking untreated | 2.7 (1.32–5.93) **†** | age (matched), residence (matched) | Case definition |
|  | Nguyen et al. (2014) (1) | Sierra Leone | Unsafe water | 3.43 (1.07–11.04) ****†** | age (matched), neighborhood (matched), education | Stool culture |
|  | Nguyen et al. (2014) (2) | Sierra Leone | Unsafe water | 4.7 (0.9–47) ***†** | age (matched), neighborhood (matched) | Stool culture |
|  | Nguyen et al. (2017) | Vietnam | Drinks boiled water: Sometimes, often or never | 2.62 (1.03–6.67) ****†** | commune (matched), sex (matched), age (matched), primary education or illiterate, lives with people who had acute diarrhea, main source of water close to a toilet, self-perceived changes in the color, odor, appearance and taste of water, drinks bottled water, drinks indoor tap water | Rectal swab |
|  | Oguttu et al. (2017) | Uganda | Not boiling water before drinking | ∞ (1–∞) * |  | Stool culture |
|  | Pande et al. (2018) | Uganda | No treatment of drinking water | 0.29 (0.099–0.82) * | age (matched) | Stool culture |
|  | Quick et al. (1995) | El Salvador | Drank untreated water | 1.9 (0.5–7.2) ***†** | age (matched), sex (matched), neighborhood (matched) | Stool culture |
|  | Reller et al. (2001) | Madagascar | Untreated water, any source | 5 (1.3–25.4) | age (matched), sex (matched), neighborhood (matched) | Stool culture |
|  | Ries et al. (1992) (1) | Peru | Drank unboiled water | 3.9 (1.7–8.9) * | age (matched), sex (matched) | Rectal swab |
|  | Ries et al. (1992) (2) | Peru | Drank unboiled water | 6.2 (1.8–20.8) * | age (matched), sex (matched) | rectal swab, blood culture |
|  | Sasaki et al.(2008) | Zambia | Not chlorinate drinking water | 2.23 (1.01–4.94) ***†** | sex (matched), age (matched), SES | Stool culture, rectal swab |
|  | Swerdlow et al. (1992) | Peru | Drank unboiled water | 3.1 (1.3–7.3) * | age (matched), sex (matched) | Rectal swab, blood culture |
|  | Weber et al. (1994) | Ecuador | Unboiled water | 3.6 (1.8–7.5) **†** | age (matched), sex (matched), neighborhood (matched | Rectal swab, blood culture |
| Water Management – Safe Storage | Beatty et al. (2004) (1) | Marshall Islands | Bucket with lid | 0.41 (0.16–1) * | sex (matched), age (matched), neighborhood (matched) | Case definition |
|  | Beatty et al. (2004) (1) | Marshall Islands | Narrow-mouthed jug with or without lid | 0.76 (0.32–1.83) * | sex (matched), age (matched), neighborhood (matched) | Case definition |
|  | Beatty et al. (2004) (1) | Marshall Islands | Insulated water cooler | 0.24 (0.09–0.58) * | sex (matched), age (matched), neighborhood (matched) | Case definition |
|  | Beatty et al. (2004) (2) | Marshall Islands | Bucket with lid | 0.41 (0.13–1.2) * | sex (matched), age (matched), neighborhood (matched) | Serologic test |
|  | Beatty et al. (2004) (2) | Marshall Islands | Narrow-mouthed jug with or without lid | 0.84 (0.27–2.69) * | sex (matched), age (matched), neighborhood (matched) | Serologic test |
|  | Beatty et al. (2004) (2) | Marshall Islands | Insulated water cooler | 0.18 (0.04–0.6) * | sex (matched), age (matched), neighborhood (matched) | Serologic test |
|  | Bhunia & Ghosh (2011) | India | Narrow-mouthed container | 0.01 (0.001–0.07) ***†** | residence (matched), race (matched), SES (matched) | Stool culture |
|  | Birmingham et al. (1997) | Burundi | Used 20 L jerry-can to store drinking water | 3.2 (1.1–9.9) ****†** | sex (matched), age (matched), residence (matched), drank lake water, bathed in lake | Stool culture |
|  | Birmingham et al. (1997) | Burundi | Clay pot to store drinking water | 0.6 (–) * | sex (matched), age (matched), residence (matched) | Stool culture |
|  | Hatch et al. (1994) | Malawi | 1 water container (closed lids, shape undisclosed) | 0.02 (0.02–0.06) |  | Stool culture, rectal swab |
|  | Hatch et al. (1994) | Malawi | 2+ water container (closed lids, shape undisclosed) | 0 (0–0.02) |  | Stool culture, rectal swab |
|  | Hatch et al. (1994) | Malawi | Any water container | 0.02 (0.003–0.12) ****†** | SES, number of children <5 years, resident in Malawi <3 months, any soap in household reside in transit center | Stool culture, rectal swab |
|  | Kirk et al. (2005) | Micronesia | Container to store water safely | 0.1 (0–0.9) | age (matched), sex (matched) | Case definition |
|  | Kirk et al. (2005) | Micronesia | Narrow-neck water container | 0.2 (0–0.8) **†** | age (matched), sex (matched) | Case definition |
|  | Nanzaluka et al. (2020) | Zambia | Water Storage - Jerrican | 1 (0.37–2.34) **†** | age (matched), residence (matched) | Case definition |
|  | O'Connor et al. (2011) | Haiti | Plastic bottle (safe storage) | 0.6 (0.2–2) * | age (matched), sex (matched) | Case definition |
|  | Quick et al. (1995) | El Salvador | Covered drinking water vessel in home | 0.2 (0–1.2) ***†** | age (matched), sex (matched), neighborhood (matched) | Stool culture |
|  | Reller et al. (2001) | Madagascar | Water stored in covered container | 3.3 (0.6–15) | age (matched), sex (matched), neighborhood (matched) | Stool culture |
|  | Shultz al. (2009) | Kenya | Storing water in jerry can | 2.8 (0.6–14.4) * | age (matched), residence (matched) | Case definition |
| Water Management – Unsafe Storage | Birmingham et al. (1997) | Burundi | Bucket to store drinking water | 4 (–) * | sex (matched), age (matched), residence (matched) | Stool culture |
|  | Cummings et al. (2012) | Uganda | Does not store water in sealed container | 3.3 (1.7–6.7) **†** |  | Stool culture |
|  | Dan-Nwafor et al. (2019) | Nigeria | Drinking water storage container without cover | 3.2 (0.3–36.1) |  | Rapid test |
|  | Dutta et al. (2021) | India | Wide neck water storage container | 0.7 (0.4–1.4) |  | Stool culture |
|  | Dutta et al. (2021) | India | Uncovered storage of drinking water | 0.9 (0.4–1.9) |  | Stool culture |
|  | Gidado et al. (2018) | Nigeria | Storing drinking water in a container without cover | 2.6 (0.49–13.8) **†** |  | Stool culture |
|  | Mahamud et al. (2012) | Kenya | Dirty water storage containers | 4,3 (1.12–17.14) ** | age (matched), residence (matched), Used soap to wash hands | Case definition |
|  | Mugoya et al. (2008) (1) | Kenya | Storing water in a bucket | 3.8 (1.2–12) ** | age (matched), neighborhood (matched), Unsound latrine superstructure, Not treating drinking water | Stool culture |
|  | Mugoya et al. (2008) (2) | Kenya | Storing drinking water in open container | 3.3 (1–10) ** | age (matched), neighborhood (matched), changing main source of drinking water, not treating drinking water, drinking water from outside the home, not washing hands with soap before eating, not washing hands after visiting toilet | Stool culture |
|  | Nanzaluka et al. (2020) | Zambia | Water Storage - Bucket | 0.4 (0.21–0.91) **†** | age (matched), residence (matched) | Case definition |
|  | Nguyen et al. (2017) | Vietnam | Self-perceived changes in the color, odor, appearance and taste of water | 1.99 (0.84–4.71) **†** | commune (matched), sex (matched), age (matched), main source of water close to a toilet, drinks boiled water: Sometimes, often or never, drinks bottled water, drinks indoor tap water | Rectal swab |
|  | O'Connor et al. (2011) | Haiti | Lacked safe water storage | 1.3 (0.5–4) * | age (matched), sex (matched) | Case definition |
|  | O'Connor et al. (2011) | Haiti | Bucket (unsafe storage) | 1.1 (0.4–2.8) * | age (matched), sex (matched) | Case definition |
|  | Rodrigues, Brun & Sandstrom (1997) | Guinea-Bissau | Bucket/basin (for water storage) | 2.81 (1.28–6.19) ** | age (matched), gender (matched), residence (matched), soap in the house, well or lake as drinking source | Case definition |
|  | Shultz al. (2009) | Kenya | Keep water stored in house covered | 0.49 (0.25–0.96) ** | age (matched), residence (matched), three or more households sharing same latrine | Case definition |
|  | Singh et al. (2020) | India | Drinking water - Storage container - uncovered | 1 (0.06–16.49) |  | Stool culture |
|  | Singh et al. (2020) | India | Drinking water - Storage container - wide mouth | 0.76 (0.33–1.75) **†** |  | Stool culture |

*adjusted odds ratio from calculating matched OR

**adjusted odds ratio from multivariate analysis

**†**these values were included in the meta-analysis

Note: SES – Socioeconomic Status

Table S2. Results from Egger’s regression.

| **Indicator of water quality** | | | **Intercept** | **95% CI** | **z** | **p** |
| --- | --- | --- | --- | --- | --- | --- |
| Improved water | | |  |  |  |  |
|  | Safely managed water | |  |  |  |  |
|  |  | Sachet water | 1.276 | -0.667–3.219 | -1.241 | 0.214 |
|  |  | Bottled water | -0.289 | -8.927– 8.348 | -0.085 | 0.932 |
|  |  | Tap water | 0.160 | -2.701– 3.021 | -0.176 | 0.861 |
|  | Basic water | | -1.812 | -3.392– -0.233 | 1.441 | 0.150 |
|  | Limited water | | -0.939 | -6.664–4.786 | 0.287 | 0.774 |
| Unimproved water | | | 0.946 | -1.167–3.060 | 0.128 | 0.898 |
| Surface Water | | | 0.171 | -1.094–1.437 | 1.677 | 0.094 |
| Treated Water | | | -0.339 | -1.925–1.246 | -0.701 | 0.483 |
|  |  | Boiled | 0.532 | -3.026–4.090 | -0.852 | 0.394 |
|  |  | Chlorinated | 0.764 | -1.231–2.758 | -1.760 | 0.078 |
|  |  | Observation of Treatment Materials | 1.249 | -0.993–3.490 | -1.890 | 0.059 |
| Untreated water | | | 0.481 | -0.234–1.196 | 1.257 | 0.209 |
| Safe water storage | | | 0.143 | -4.209–4.494 | -0.898 | 0.369 |
| Unsafe water storage | | | -0.406 | -3.119–2.306 | 0.526 | 0.599 |

*p < 0.05

**p < 0.01
